# Supplementary material for: Endometrial cell-derived small extracellular vesicle miR-100-5p promotes functions of trophoblast during embryo implantation
Source: Mol Ther Nucleic Acids. 2020 Nov 5;23:217–31. doi: 10.1016/j.omtn.2020.10.043 (PMC7758458; doi:10.1016/j.omtn.2020.10.043)
Supplement: Document S1. Figures S1–S5 and Table S2 [file mmc1.pdf]

OMTN, Volume *23*

Supplemental Information

Endometrial cell-derived small extracellular vesicle miR-100-5p promotes functions of trophoblast during embryo implantation

Qiang Tan, Shuang Shi, Jingjie Liang, Dingren Cao, Shaoyu Wang, and Zhengguang Wang

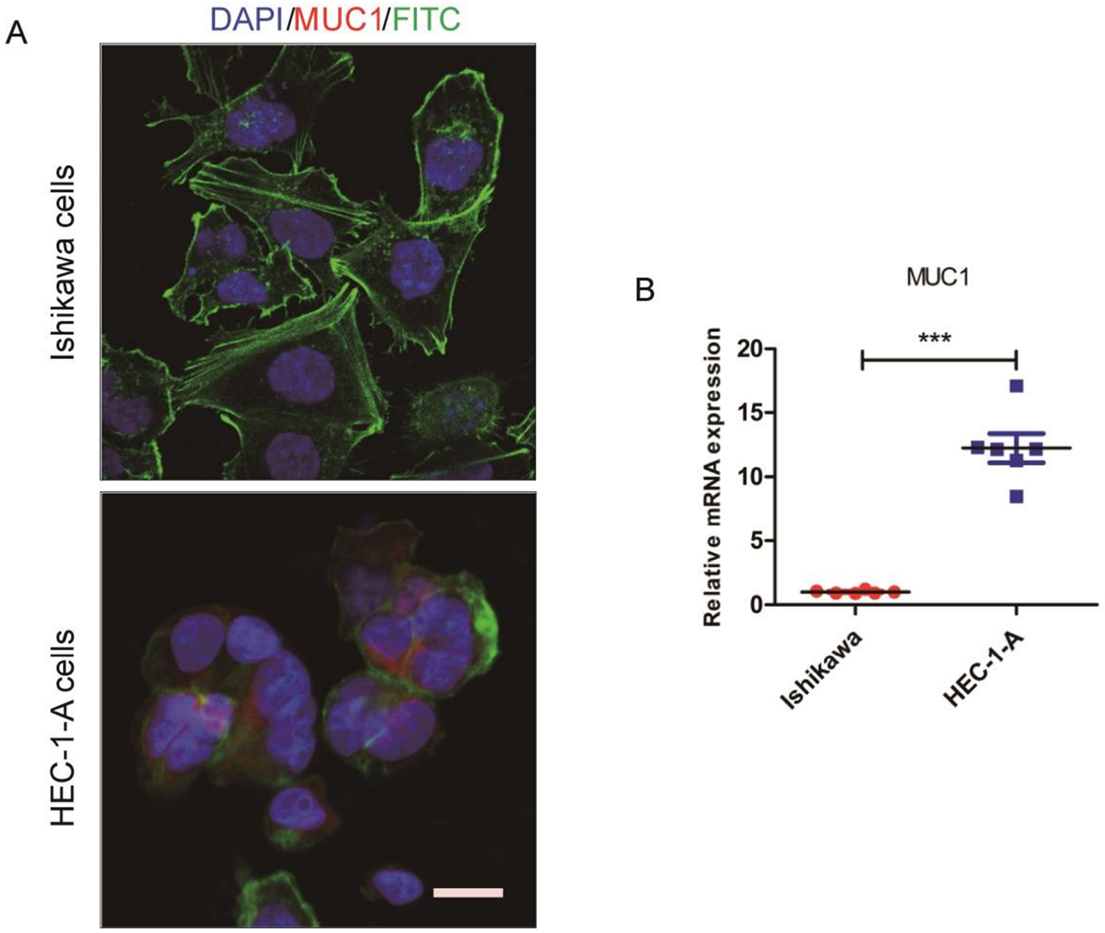

Figure S1. Ishikawa cells and HEC-1-A cells represent receptive and non-receptive endometrium epithelial cells, respectively. (A) Immunofluorescence image of Ishikawa and HEC-1-A cells stained with an MUC1 antibody, a molecular marker of uterine receptivity. Nuclei stained using DAPI dye. FITC-conjugated phalloidin was used to label the cytoskeleton. The scale bar = 20μm. (**B**) The mRNA expression level of MUC1 in Ishikawa and HEC-1-A cells was detected by qRT-PCR. (**P*<0.05; ***P*<0.01; ****P*<0.001)

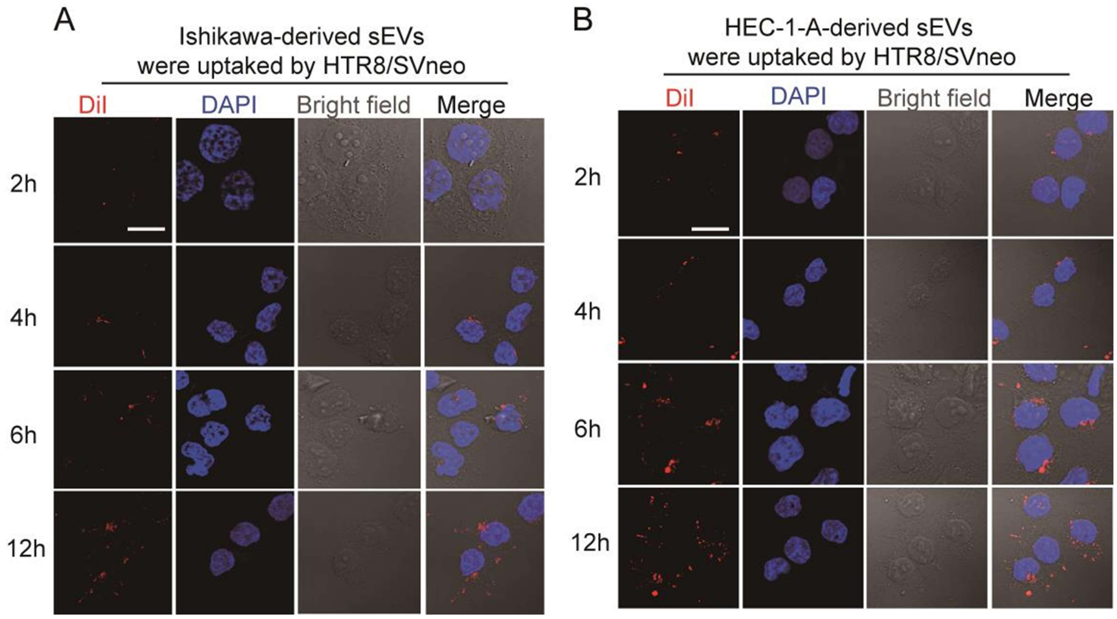

Figure S2. sEVs derived from endometrium epithelial cells could be uptake by HTR8/SVneo trophoblasts. sEVs were labeled using DiI dye and incubated with serum-starved trophoblasts for 2, 4, 6 and 12h, respectively. Confocal microscopic images showed uptake of sEVs derived from Ishikawa cells (**A**) and HEC-1-A cells (**B**) by HTR8/SVneo cells in a time-dependent manner. Nuclei stained by DAPI in blue. The scale bar = 20μm.

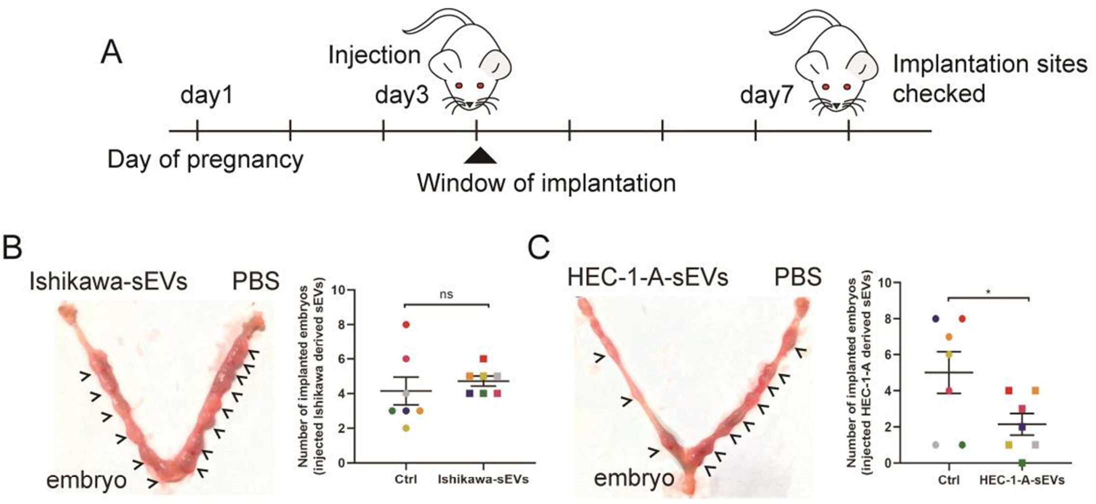

Figure S3. Endometrial epithelial cells derived sEVs impair embryo implantation. (A) Schematic diagram showed the female mouse after mating were injected on day3 of pregnancy, and the number of implanted embryos were checked on day7 of pregnancy. (**B-C)** The number of implanted embryos after injecting Ishikawa or HEC-1-A derived sEVs. PBS was used as a control. (ns: no significant. **P*<0.05; ***P*<0.01; ****P*<0.001)

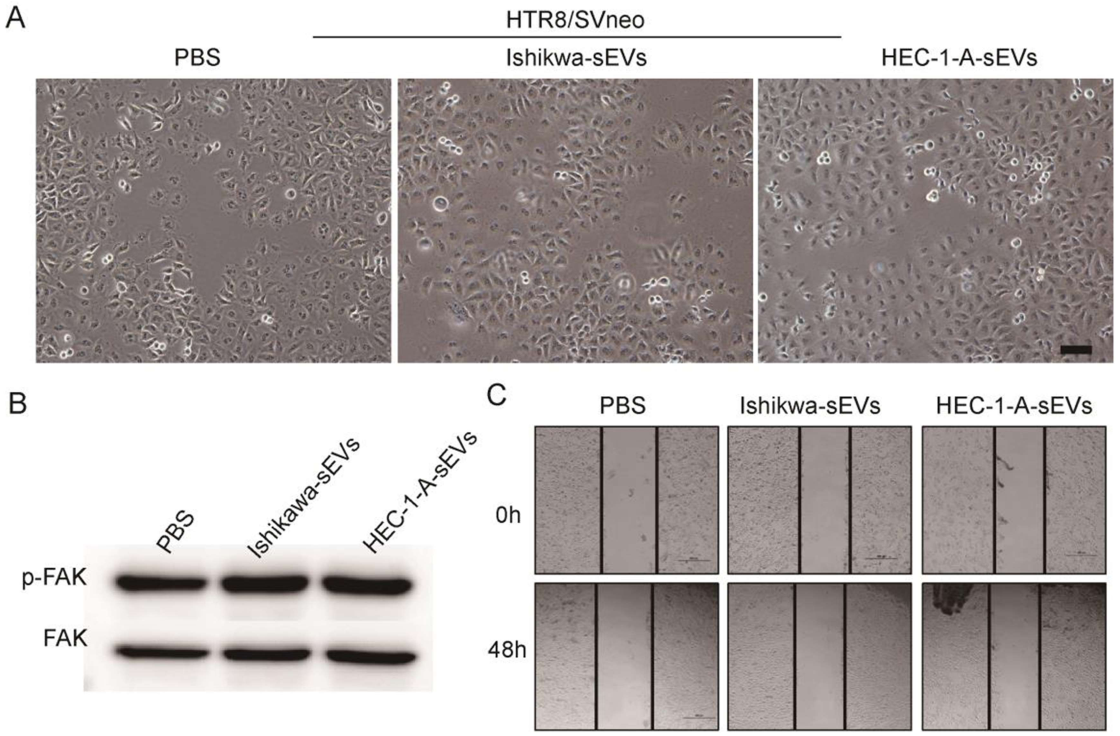

Figure S4. Growing state of HTR8/SVneo cells after stimulation with PBS, Ishikawa- sEVs, or HEC-1-A- sEVs for 24h. (A) The images visualized using the optical microscope. The scale bar = 50μm. (**B**) HTR8/SVneo trophoblasts were serum-starved and treated with PBS, 50μg Ishikawa-sEVs or 50μg HEC-1-A-sEVs. After 48h, the cells were immunoblotted for phosphorylated FAK (P-FAK). (**C)** Images of wound closure assays performed on HTR8/SVneo cells cultured in serum-free medium supplemented PBS, 50μg Ishikawa-sEVs or 50μg HEC-1-A-sEVs for 48h.

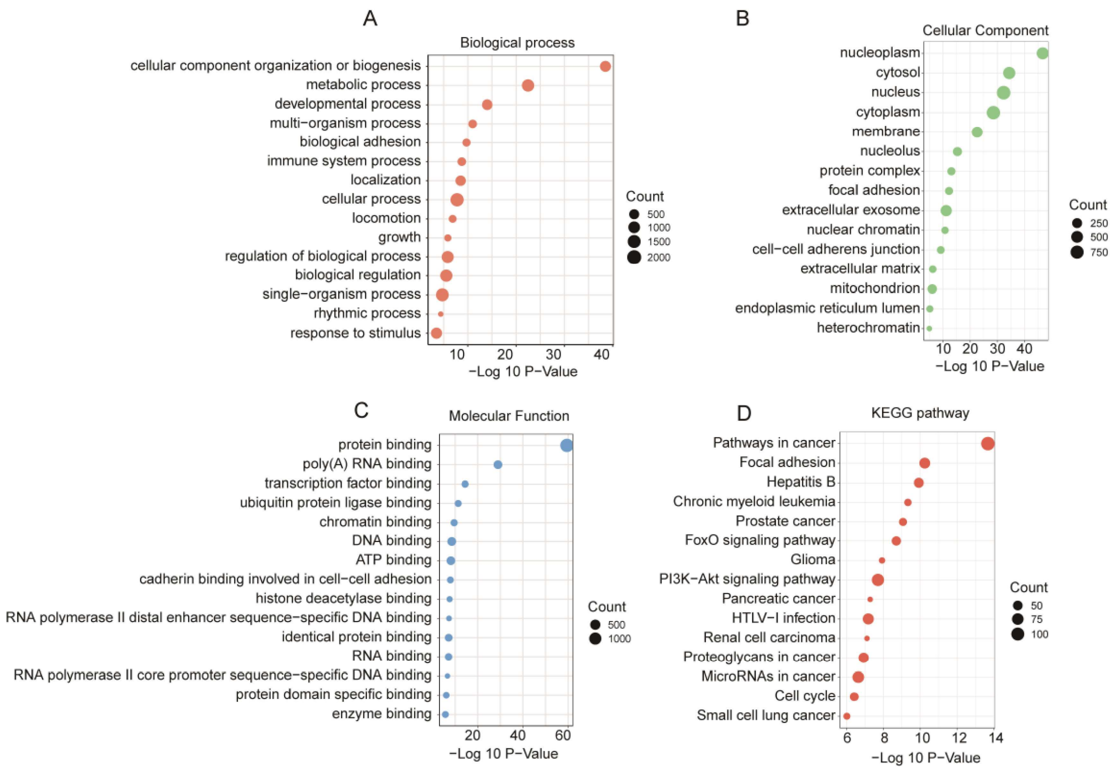

Figure S5. GO and KEGG analysis of genes, which regulated by the differentially expressed miRNAs between Ishikawa-sEVs and HEC-1-A- sEVs. (A-C) A bubble plot of GOs (Biological Process, Molecular function and Cellular components) enriched. (**B**) A bubble plot of KEGG pathway enriched.

Table S1 MiRNA expression profile in endometrial cells derived exosomes (Ishikawa-sEVs vs. HEC-1-A-sEVs)

Table S2 Sequences of primers, agomir, and miRNA mimics.

MUC1	F: 5'TGCCGCCGAAAGAACTACG3'
	R: 5'TGGGGTACTCGCTCATAGGAT3'
GAPDH	F: 5'GGAGCGAGATCCCTCCAAAAT3'
	R: 5'GGCTGTTGTCATACTTCTCATGG3'
miR-100-5p	5'AACCCGTAGATCCGAACTTGTG3'
cel-miR-39	5'TCACCGGGTGTAAATCAGCTTG3'
U6	5'AACGAGAAGCGAACCAAAAAAA3'
miR-100-5p(antagomir)	5'CACAAGUUCGGAUCUACGGGUU3'
miR-100-5p(mimics)	5'AACCCGUAGAUCCGAACUUGUG3'
	5'CAAGUUCGGAUCUACGGGUUUU3'
Negative control (mimics)	5'UUCUCCGAACGUGUCACGUTT3'
	5'ACGUGACACGUUCGGAGAATT3'
