# Supplementary material for: Endometrial cell-derived small extracellular vesicle miR-100-5p promotes functions of trophoblast during embryo implantation
Source: Mol Ther Nucleic Acids. 2020 Nov 5;23:217–31. doi: 10.1016/j.omtn.2020.10.043 (PMC7758458; doi:10.1016/j.omtn.2020.10.043)
Supplement: Document S2. Article plus supplemental information [file mmc3.pdf]

Molecular Therapy
Nucleic Acids
Original Article

Endometrial cell-derived small extracellular vesicle miR-100-5p promotes functions of trophoblast during embryo implantation

Qiang Tan,[1,3] Shuang Shi,[1,3] Jingjie Liang,[1] Dingren Cao,[1] Shaoyu Wang,[1] and Zhengguang Wang[1,2]

[1]College of Animal Science, Zhejiang University, Hangzhou 310058, PR China; [2]Hainan Institute of Zhejiang University, Sanya 572000, PR China

Communication between maternal uterus and blastocyst occurs in the early stages of pregnancy, and the interaction influences the success of embryo implantation. Whereas small extracellular vesicles (sEVs) play an essential role in mediating intercellular communication in numerous biological processes, their role in embryo implantation during the window of implantation (WOI) remains poorly defined. Here, we report that endometrial epithelial cells (EECs) secrete sEVs during early pregnancy, which affects the trophoblast behaviors (migration, invasion, and proliferation), thus influencing embryo implantation. We show that microRNA (miR)-100-5p, sEVs containing microRNA (miRNA), activates both focal adhesion kinase (FAK) and c-Jun N-terminal kinase (JNK), as well as contributes to trophoblast migration and invasion. Furthermore, our findings indicate that the sEV miR-100-5p promotes angiogenesis during the implantation process. In conclusion, this study reveals a novel mechanism by which EEC-derived sEV miR-100-5p crosstalks with trophoblasts, leading to an enhanced ability for implantation.

INTRODUCTION

Normal pregnancy depends on the successful implantation. Embryo implantation is a complex and critical process that demands synchronous communication between the maternal uterus and blastocyst.[1] One of the essential early pregnancy periods is the window of implantation (WOI), a period that is primarily under the direction of ovarian estrogen (E2) and progesterone (P4).[2] The uterus achieves receptivity during this period. In humans, the receptivity of the uterus lasts for approximately 4 days in the mid-secretory phase of the menstrual cycle.[3] In mice, the period of uterine receptivity occurs from late on day 3 (D3) to the morning of D4 of pregnancy.[4] During the WOI, the endometrium gradually loses the polarity of the epithelial cells, and embryos initiate the interplay with maternal endometrium.[5] During the establishment of implantation, the trophectoderm layer of the blastocyst attaches to the endometrial epithelium, and the trophoblasts migrate and invade the maternal uterus. Thereafter, the trophoblasts proliferate to create the placenta, accompanied by angiogenesis.[2,6,7] The uterus undergoes significant morphological and molecular changes in this early stage of pregnancy, and these changes contribute to the acquisition of receptivity prior to blastocyst attachment.[4] The molecular information exchanges between uterus and embryos determine successful pregnancy during WOI. Evidence from previous studies suggests that errors in embryo implantation can result in poor outcome, such as spontaneous abortions and other pregnancy diseases.[8] Hence, understanding the communication and events in embryo implantation is essential for a healthy pregnancy.

Extracellular vesicles (EVs) play a central role in the mediation of cell-cell or cell-environment communication. Almost all types of cells, ranging from germ cells to tumor cells, generate and secrete EVs.[9–12] Exosomes are EVs that range in size from 40 to 200 nm and are derived from multivesicular bodies (MVBs) in the early endosomal compartment. The exosomes are released into extracellular space when MVBs fuse with the plasma membrane,[13,14] but studies considered that a significant amount of small EVs (sEVs) were regarded as exosomes when the source of EVs derived using ultracentrifugation. Recent cumulative studies suggest that exosomes play crucial roles as mediators of intercellular communication, as well as act as biomarkers.[15–17] Exosomes have received a lot of research attention because of the kind and importance of the cargos they carry. The exosomal cargo includes cell surface receptor proteins, microRNAs (miRNAs), extracellular matrix proteins, and lipids.[13,14,18] Furthermore, delivery of this exosomal content leads to both phenotypic and functional changes in the recipient cells.[16] Exosomal cargos, especially exosomal miRNAs, have been extensively studied in tumor progression, where they have been shown to promote cell migration and invasion.[10,19] During WOI, the trophoblasts behave similar to tumor cells in relation to migration, growth, and invasion.[20] Other studies have revealed that miRNAs are mainly enriched in exosomes and involved in embryo implantation.[21,22] Recently, more and more studies have pay attention to the role of exosomal miRNAs in embryo implantation. Exosomal hsa-microRNA (miR)-30d, secreted by human endometrium, modifies the transcriptome of the preimplantation embryo, therefore affecting embryo attachment.[23] Even sEVs contained with miRNAs from maternal endometrium regulate

Received 16 July 2020; accepted 30 October 2020;
https://doi.org/10.1016/j.omtn.2020.10.043.

[3]These authors contributed equally

Correspondence: Zhengguang Wang, College of Animal Science, Zhejiang University, Hangzhou 310058, PR China.
E-mail: wzhguang68@zju.edu.cn

This is an open access article under the CC BY license (http://creativecommons.org/licenses/by/4.0/).

embryo development.[24] However, literature on the mechanism of action of the uterus-derived exosomal miRNAs in embryo implantation during WOI is scant.

Besides, cellular chemical signaling pathways, such as autocrine, paracrine, or endocrine, play major roles during embryo implantation. Alongside the EVs, the maternal uterus secretes various factors into the uterine cavity during embryo implantation. Here, we embarked on exploring how the sEVs, which are derived from receptive endometrial cells during WOI, mediate intercellular communications and their involvement in promoting implantation. We show that receptive endometrial epithelial cells (EECs) release more sEVs than nonreceptive cells. These sEVs can activate the signaling pathways in trophoblasts, thus promoting the migration and invasion, which affect implantation rates. Interestingly, we identify the critical EEC-derived sEV miRNA, which enhances the implantation ability of trophoblasts. Finally, the findings show that sEV-derived miRNAs are involved in angiogenesis, thus influencing successful implantation. Our study provides a novel intercellular communication mechanism during embryo implantation.

RESULTS

Endometrial cells generate and secrete sEVs

The WOI is a critical period for successful implantation. The WOI defines uterus receptivity and allows adherence of blastocysts.[4] Mucin 1 (MUC1), a transmembrane glycoprotein expressed on the luminal epithelium, was considered to be a significant indicator of uterine receptivity.[25] The expression of MUC1 decreases when the uterus achieves receptivity. In this study, Ishikawa or HEC-1-A cell lines were selected as receptive or nonreceptive epithelial cells, respectively. The Ishikawa cell line supports embryo attachment and is widely considered to be a good model for normal endometrium study, whereas the HEC-1-A has low adhesive properties and is generally used as a model of nonreceptive epithelial cells.[26] Indeed, we observed high expression of MUC1 in HEC-1-A cells but not in Ishikawa cells (Figures 1A, S1A, and S1B). It has been reported that during embryo implantation, the endometrium secretes soluble factors, such as proteins and miRNAs, into the uterine cavity.[23] Whereas these molecules are secreted into the cavity, their crosstalk between maternal endometrium and embryo during this period is yet to be defined. To retain the vitality of the cells, we stained Ishikawa or HEC-1-A cells with DiI dye. After washing in phosphate-buffered saline (PBS), the labeled cells were coincubated with HTR8/SVneo cells using a Transwell chamber with a 0.3-μm membrane pore (Figure 1B). The appearance of red fluorescent DiI dye in HTR8/SVneo demonstrated that the secretion was delivered from EECs in the upper well to the recipient trophoblast cells seeded in the lower well (Figure 1B). To gain further insight into the secretion of exosomes, an electron microscopic was employed to investigate the number and morphology of intraluminal vesicles (ILVs) and MVBs in EECs (Figure 1C). Interestingly, the number of MVBs per cell and the number of ILVs per MVB dramatically increased in Ishikawa cells compared with HEC-1-A cells, indicating that the Ishikawa cells had a stronger secretory ability (Figures 1D and 1E). However, there was no morphological difference in ILVs between the two cell lines (Figure 1C).

To examine whether Ishikawa or HEC-1-A cells can secrete exosomes, cells were cultured in medium supplemented with 10% exosome-free fetal bovine serum (FBS) for 48 h. Dead cells and debris were removed from supernatants, and then the supernatant was filtered in a 0.22-μm pore-size filter to remove microvesicles larger than 200 nm in diameter (Figure 2A). Exosomes are heterogeneous vesicles and range from 40 to 200 nm in diameter.[14] Both nanoparticle tracking analysis (NTA) (Figure 2B) and electron microscopy (Figures 2C and 2D) revealed that particles isolated using our method contain abundant and typical sEVs. Among these sEVs, a significant amount of them were considered to be exosomes. Whereas there was a significant difference in the mean particle size of sEVs derived from the two cell lines, probably mainly because of uneven distribution of sample particles, there was no difference in total protein content. On the other hand, the concentration of Ishikawa-sEVs was higher compared to HEC-1-A-sEVs (Figure 2E). The sEV marker proteins, such as CD63, CD9, Alix, TSG101, or heat shock protein (HSP)70, were evaluated by immunoblot analysis of the sEVs compared to the whole cell lysates (WCLs), as shown in Figure 2F. The sEV proteins were primarily detected in sEVs. Unlike the WCL, neither the Ishikawa- nor the HEC-1-A-derived sEVs reacted with calnexin (CANX), an endoplasmic reticulum protein marker. In addition, we detected CD63, Alix, TSG101, or CANX in pellets that precipitated in each step of centrifugation (Figure 2G), suggesting that we collected purified sEVs free from cell debris contamination. Previous studies had reported that sEVs derived from the endometrium could mediate the communication between endometrial cells and embryonic trophoblast cells.[27] In our study, we tested whether the Ishikawa-sEVs or the HEC-1-A-sEVs can be taken up by trophoblast cells (HTR8/SVneo) (Figure 2H). These sEVs were labeled with the DiI dye and added into the culture medium containing HTR8/SVneo cells. Immunofluorescence (IF) results showed that both DiI-stained Ishikawa- and HEC-1-A-sEVs were incorporated into HTR8/SVneo cells, which increased with prolonged incubation time (Figures S2A and S2B). Overall, these results demonstrate that ECCs (Ishikawa or HEC-1-A) could secrete sEVs and are efficiently transported into recipient cells.

Receptive endometrium cell-derived sEVs enhance potency of trophoblast

Since the sEVs isolated from EECs are uptaken by trophoblast (HTR8/SVneo), we speculated that these sEVs might regulate the function of trophoblast, such as migration and signaling pathway. We examined the amount of the sEV protein, and the same amount of Ishikawa-sEVs or HEC-1-A-sEVs were used to treatment trophoblast cells (Figure S4A). After treatment with PBS, Ishikawa-sEVs (50 μg or 100 μg), or HEC-1-A-sEVs (50 μg or 100 μg), respectively, HTR8/SVneo cells were collected and detected the phosphorylated (P; activated) and total forms of signaling proteins widely implicated in promoting cell migration and adhesion, including focal adhesion kinase (FAK) and c-Jun N-terminal kinase (JNK). Results revealed that

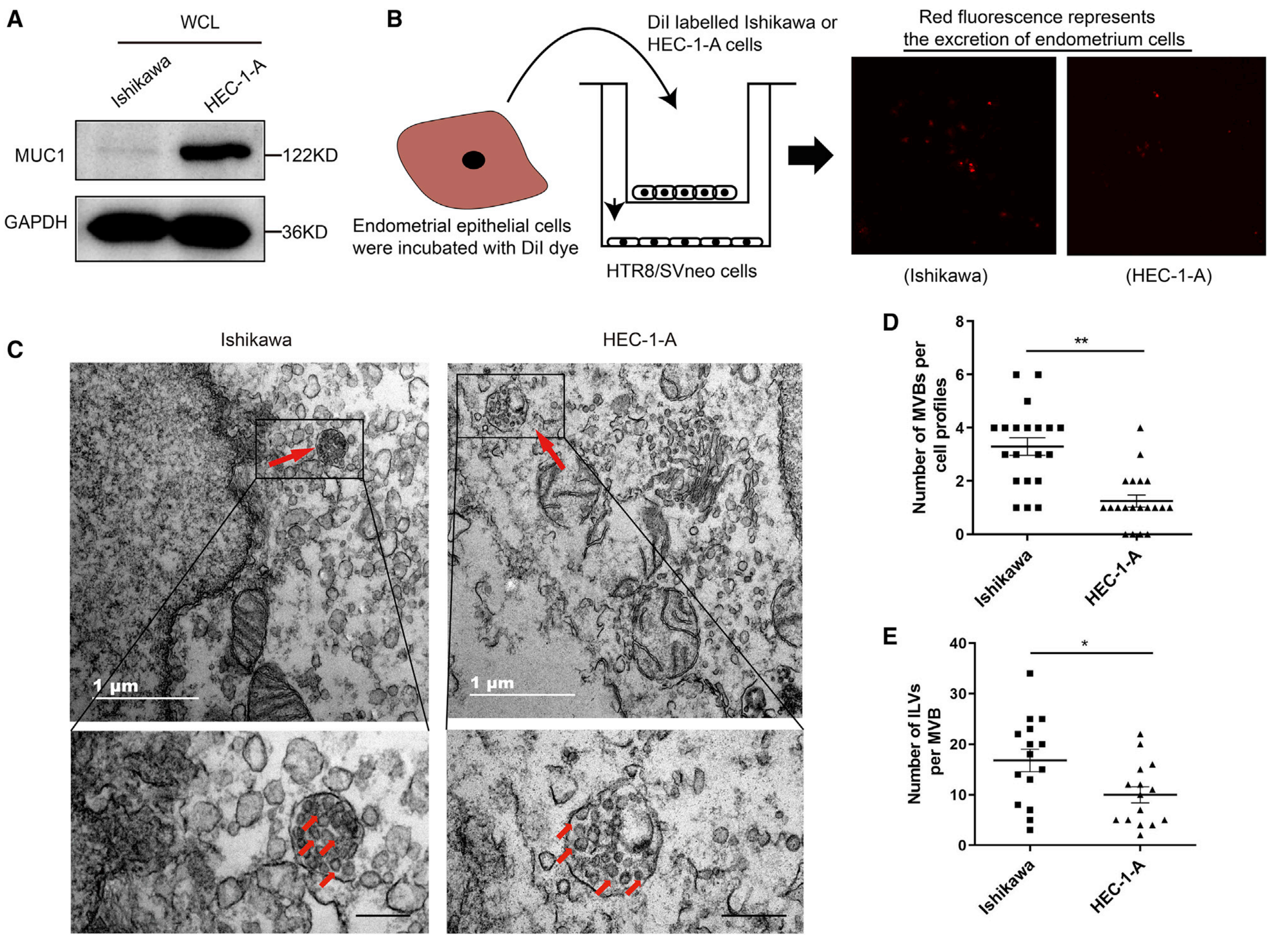

Figure 1. Endometrial cells secrete exosomes

(A) Whole cell lysates (WCLs) of Ishikawa and HEC-1-A cells were immunoblotted for the receptive markers MUC1 and GAPDH as a loading control. (B) DiI-labeled endometrial epithelial cells were coincubated with HTR8/SVneo cells in a Transwell plates (membrane pore = 0.3 μm). (C) Representative electron microscopic images Ishikawa and HEC-1-A cell lines. Scale bars, 500 nm. Red arrows in image indicate MVBs containing typical ILVs. (D) The number of MVBs per cell profile. (E) The number of ILVs per MVBs. The number of MVBs and ILVs was counted randomly, and only MVBs containing typical ILVs were counted. The results were plotted as dot plots (*p < 0.05; **p < 0.01; ***p < 0.001).

whereas 100 μg of Ishikawa-sEVs enhanced P-FAK (Figures 3B and S4B), no P-JNK was affected. There was no significant phosphorylation difference between HEC-1-A-sEV treatment and the control cells, as shown in Figures 3B and 3C. To further interrogate the effect of sEVs during implantation, we performed an injection test to examine the number of implanted embryos. The same amount (also same volume) of sEVs and PBS was injected into each side of the mouse uterine horn (left and right) in preimplantation on D3 of pregnancy, respectively (Figure S3A). We found that Ishikawa-derived sEVs increased the chances of implantation but not significantly compared to the PBS control group (Figures S3B and S3C). Conversely, the injection of HEC-1-A (nonreceptive)-derived sEVs significantly decreased the chances of implantation (Figures 3D–3F). This observation was associated with opposing embryonic adhesion that occurs during the preimplantation period.

Leading edges are considered to be a marker of polarized cell migration.[28] We examined whether sEVs derived from EECs could trigger the formation of leading edges in the HTR8/SVneo trophoblast. Trophoblasts were serum starved and incubated with PBS or 100 μg sEVs for 24 h, and then the filamentous actin (F-actin), located in leading edges of migrating cells, was stained. The fluorescence microscopy images of HTR8/SVneo showed that Ishikawa-sEV-treated trophoblasts exhibited a polarized morphology, with the F-actin localized in the leading edge of the cells (Figure 3D, middle panel). On the contrary, PBS or HEC-1-A-sEV-treated trophoblasts displayed low polarity with a round shape (Figure 3D, left and right panels). Approximately 47% of Ishikawa-sEV-treated trophoblasts showed leading edges, a two-fold increase compared with either PBS or HEC-1-A-sEV-treated HTR8/SVneo (Figure 3E). Formation of leading edges by the sEV-treated trophoblasts indicates that the sEVs derived

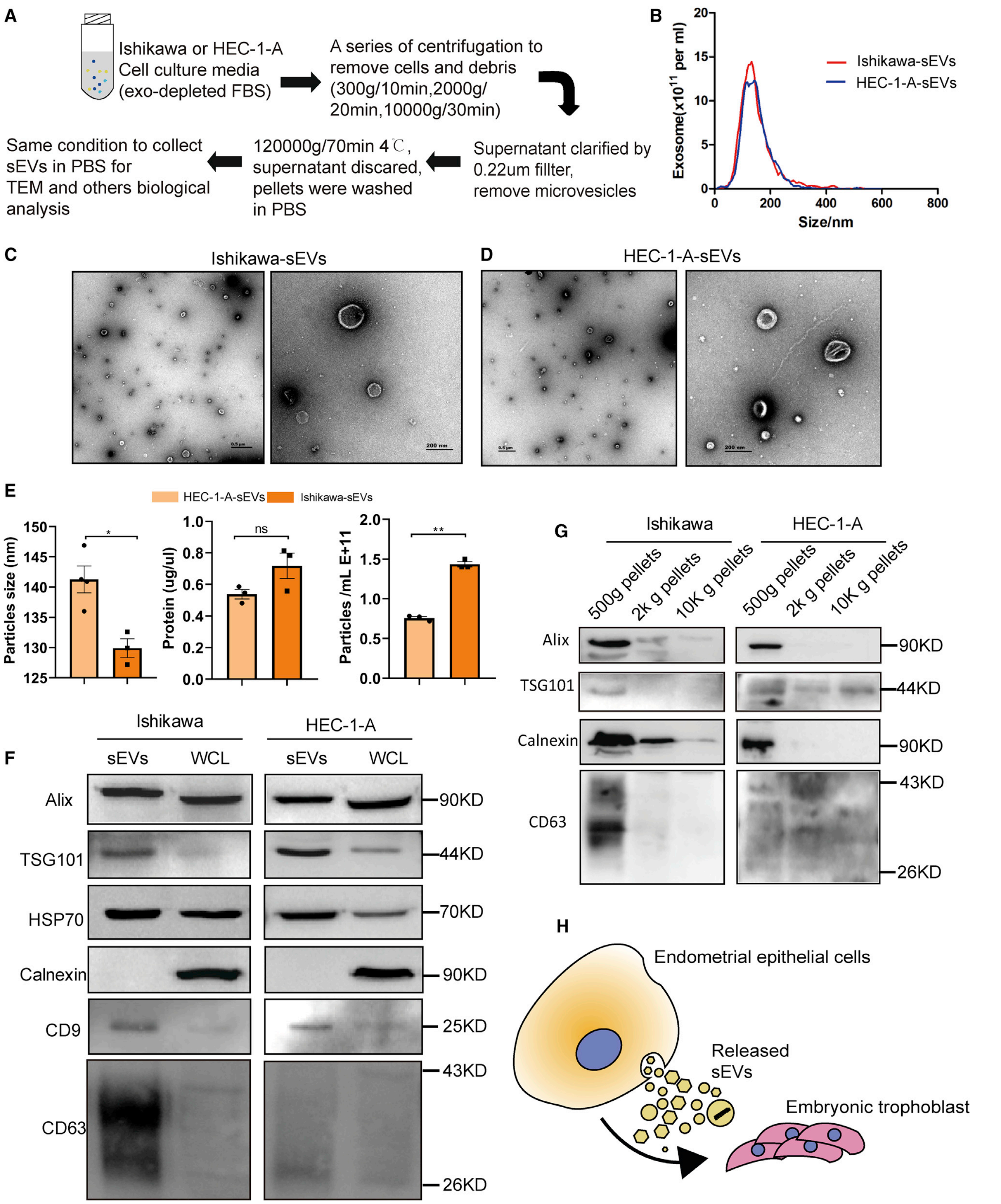

(legend on next page)

from receptive EECs promote the migration ability of trophoblasts. To further investigate the effects of sEVs in the motility potential of trophoblasts, a Transwell migration and invasion assay was performed. The results showed that Ishikawa-derived sEVs enhanced migration of trophoblasts (Figures 3F and 3G). However, PBS or HEC-1-A-sEV-treated HTR8/SVneo cells exhibited low motility potential. The same results were shown when migration was measured in wound-healing (migration) assays. The monolayers of HTR8/SVneo trophoblasts were struck to create wounds and then cultured in medium supplemented with PBS, Ishikawa-sEVs, or HEC-1-A-sEVs. After 48 h, trophoblasts with 100 μg Ishikawa-sEV stimulation were capable of migrating into the wound, whereas there was no difference in the migration of trophoblasts stimulated with PBS or HEC-1-A-sEVs or 50 μg sEV stimulation (Figures 3I, 3J, and S4C).

The invasion and proliferation of embryonic trophoblast cells have the ability for successful embryo implantation and placenta formation. These events increase the connection between the embryos and the maternal uterus for implantation. The invasion ability of HTR8/SVneo was measured by the Transwell assay. HTR8/SVneo trophoblasts were pretreated with PBS, Ishikawa-sEVs, or HEC-1-A-sEVs and then cultured in a Transwell plate coated with Matrigel. Our results showed that Ishikawa-sEVs stimulated the invasion ability of HTR8/SVneo more than the HEC-1-A-sEVs or control PBS (Figures 3F–3H). In addition, we used the 5-ethynyl-2′-deoxyuridine incorporation (EdU) assay to evaluate the effects of sEVs on trophoblasts proliferation. The measurements were conducted 24 h after the transfer of sEVs or PBS into the HTR8/SVneo cells. Whereas PBS or HEC-1-A-sEVs had no significant effect on HTR8/SVneo trophoblast proliferation, receptive EEC-derived sEVs (Ishikawa-sEVs) significantly promoted proliferation of HTR8/SVneo (Figures 3K and 3L). Collectively, the results suggest that receptive endometrium cell-derived sEVs enhance the potency of trophoblasts for embryo implantation.

sEV-derived miRNAs mediate embryo implantation

Exosomes consider being miRNA-abundant EVs, which are released by cells and delivered into recipient cells to involve the biological processes.[21] In this study, we have proven that sEVs derived from EECs affect the migration and invasion of trophoblast cells. To assess the changes in miRNA expression between Ishikawa-sEVs and HEC-1-A-sEVs during early pregnancy, we conducted the next deep sequencing of small RNAs from nonreceptive and receptive cell sEVs. There were over 300 miRNAs identified in EEC-derived sEVs (Table S1). In addition, we identified a set of miRNAs that were differentially expressed in Ishikawa versus HEC-1-A cell-derived sEVs. Among these differentially expressed miRNAs (DEMs), Figure 4A shows the most significant (with conditions: |log fold change [FC]| > 1.0 and a p value < 0.05) difference between Ishikawa and HEC-1-A cell-derived sEVs. To reveal the potential functions of the differentially expressed sEV miRNAs, we predicted the target genes of the thirteen miRNAs *in silico*. The results of bioinformatics analysis revealed that 2,405 mRNAs were potentially targeted by these miRNAs. The biological processes and signal pathways of target genes were mainly enriched in cellular process, biological adhesion, extracellular exosome, focal adhesion, and phosphatidylinositol 3-kinase (PI3K)-Akt signaling pathways (Figures S5A–S5D). The results indicate that the sEV miRNAs derived from endometrial cells might regulate the functions of trophoblast.

Given that, miR-100-5p was previously reported as being essential for cellular migration and viability. Interestingly, the miR-100-5p was significantly upregulated in villus and increased in serum during pregnancy after embryo transfer.[29] Among the 13 DEMs, miR-100-5p was especially noted. In our results, the expression level of miR-100-5p was over 20-fold higher in Ishikawa cells compared to HEC-1-A cells (Figure 4B), which led to the abundance of the miR-100-5p present in Ishikawa-derived sEVs (Figure 4C). We also compared the expression level of miR-100-5p between Ishikawa cells and Ishikawa-sEVs. The results showed that miR-100-5p was enriched in sEVs, showing that the miR-100-5p was secreted from Ishikawa cells and packaged into sEVs (Figure 4D). The high expression of miR-100-5p in receptive EECs indicated that miR-100-5p is involved the process of embryo implantation during the WOI. Our small RNA sequencing results from mice endometrium samples during early pregnancy showed that miR-100-5p increased in the endometrium on D4 pregnancy, the receptive period of the uterus (Figure 4E). Consistent with the sequencing results, the miR-100-5p was markedly increased in D3 or D4 compared to D1, D2, or D5 in mouse endometrium in the early stage of pregnancy (Figure 4F). These results indicate that miR-100-5p plays a critical role during embryo implantation. Incubation of HTR8/SVneo cells with Ishikawa cell-derived sEVs led to a significant increase in miR-100-5p content in HTR8/SVneo, whereas treatment with HEC-1-A-sEVs or PBS led to nonsignificant (ns) effects (Figure 4G). Taken together, these findings indicate that receptive endometrial cell-derived sEVs contain miR-100-5p and might mediate the communication between the embryo and maternal endometrium.

miR-100-5p is essential for embryo implantation

We next want to determine whether the EEC-derived exosomal miRNAs mediate the intercellular communications between uterus

Figure 2. Characterization of sEVs derived from endometrium epithelial cells

(A) Procedure for isolating sEVs from Ishikawa and HEC-1-A cell culture supernatant using ultracentrifugation. (B) NTA (concentration and size distribution) of Ishikawa-sEVs and HEC-1-A-sEVs. (C and D) Ishikawa-sEVs (C) and HEC-1-A-sEVs (D) were negatively stained, and representative transmission electron microscopy (TEM) images were shown, respectively. (E) Mean particle size of sEVs, protein concentration per milliliter, and particle number per milliliter. (F) Western blot analysis of WCL and exosomal protein markers Alix, TSG101, HSP70, CD9 and CD63, and calnexin as a negative control. (G) The pellets precipitated in each step of differential centrifugation were blotted for exosomal marker CD63, Alix, TSG101, and endoplasmic reticulum marker calnexin. (H) Schematic of *in vitro* functional assay of endometrial cell sEVs. sEVs derived from endometrial epithelial cells carry cargos and deliver to embryonic trophoblast cells (*p < 0.05; **p < 0.01; ***p < 0.001).

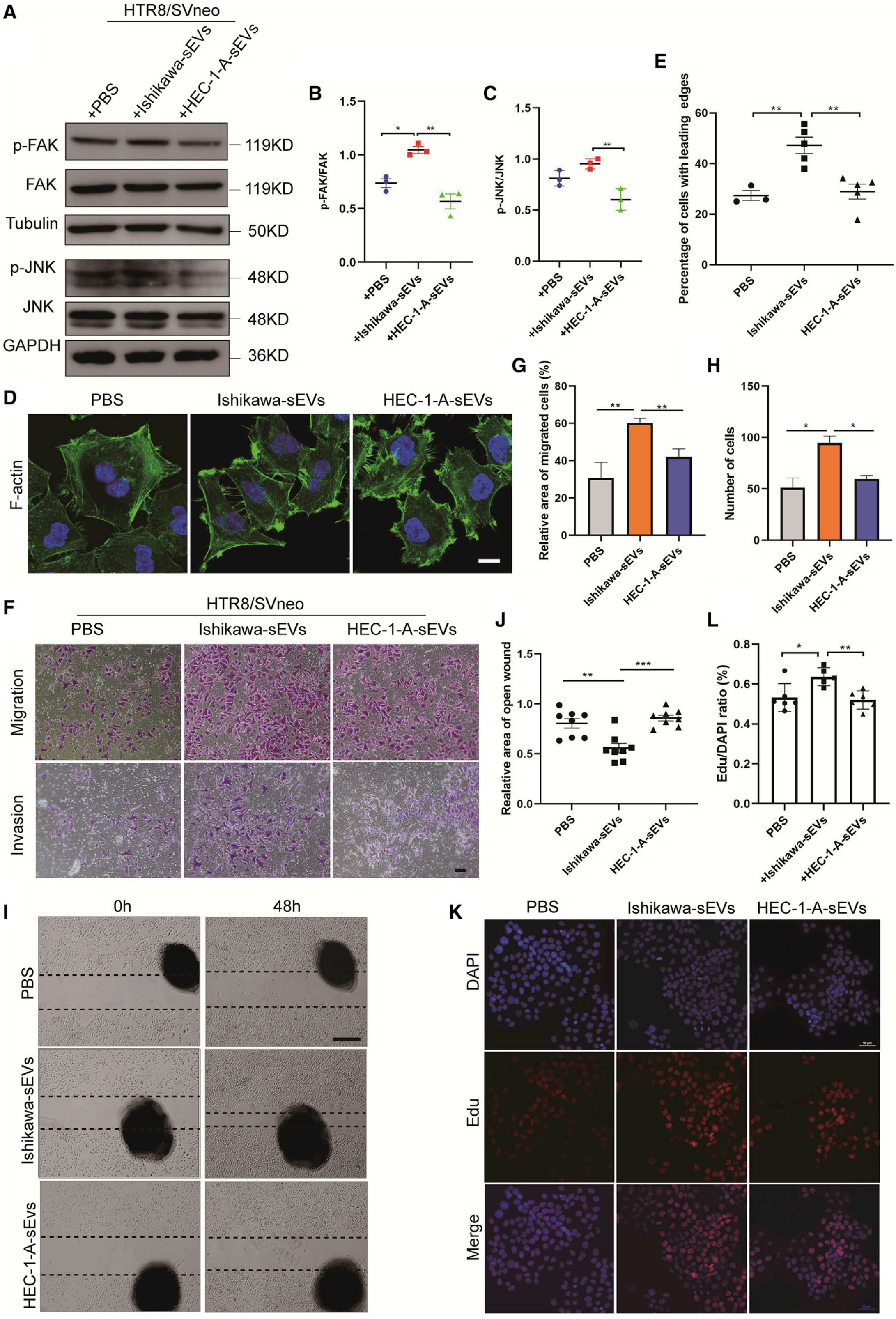

(legend on next page)

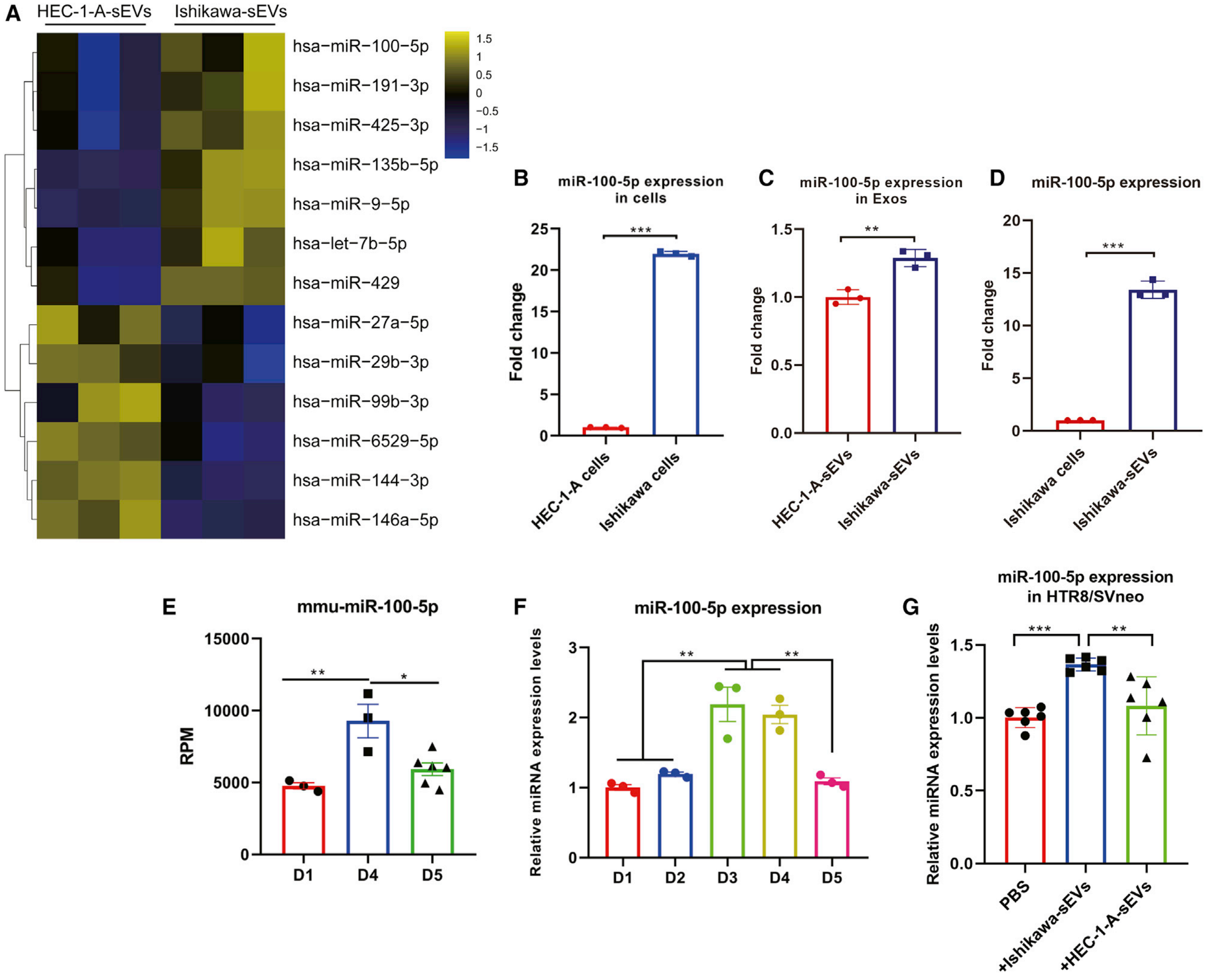

Figure 4. The miRNA profiles of endometrial cell-derived sEVs

(A) Differential expressed miRNA profiles between Ishikawa- and HEC-1-A-derived sEVs. (B) Expression level of miR-100-5p in Ishikawa and HEC-1-A cells. (C) miR-100-5p abundance in sEVs secreted by Ishikawa and HEC-1-A. (D) Comparison of the miR-100-5p expression level in Ishikawa and sEVs released by Ishikawa. (E) The fold change of miR-100-5p in mouse endometrium on pregnancy D1, D4, and D5. (F) The relative expression of miR-100-5p in mouse endometrium in the early stage of pregnancy. (G) After treatment with PBS, Ishikawa-sEVs, and HEC-1-A-sEVs for 24 h, respectively, the level of miR-100-5p in HTR8/SVneo was measured by qRT-PCR (*p < 0.05; **p < 0.01; ***p < 0.001).

and embryos. Besides, we explored whether the exosomal miRNAs affect the function of trophoblasts during embryo implantation. We first determined the effect of miR-100-5p in embryo implantation by injecting miR-100-5p antagomir or diethyl pyrocarbonate (DEPC) water into the female uterine horn on D3 of pregnancy. The implantation sites were checked on D7 of pregnancy. As shown

Figure 3. Endometrial epithelial cell-derived sEVs affect embryo implantation

(A–C) HTR8/SVneo trophoblasts were serum starved and treated with PBS, Ishikawa-sEVs, or HEC-1-A-sEVs. After 48 h, the cells were immunoblotted for phosphorylated FAK (P-FAK) and P-JNK. The blots were also detected for total FAK, JNK, and tubulin or GAPDH. The ratio of phospho protein was measured and calculated using phospho protein/total protein. (D) HTR8/SVneo trophoblasts were incubated with serum-free medium supplemented with PBS, Ishikawa-sEVs, or HEC-1-A-sEVs for 12 h, and then the HTR8/SVneo cells were stained for F-actin using FITC-conjugated phalloidin. The white arrows show leading edges. Bar, 20 μm. (E) The percentages of cells with leading edges were determined. (F) Migration and invasion assay of HTR8/SVneo cells treated with PBS, Ishikawa-sEVs, or HEC-1-A-sEVs using Transwell. Bar, 50 μm. (G and H) Migrated and invaded cells were counted, and representative images were shown. (I) Images of wound-closure assays performed on HTR8/SVneo cells cultured in serum-free medium supplemented with PBS, Ishikawa-sEVs, or HEC-1-A-sEVs for 48 h. The dashed lines indicate the width of the wound. Bar, 500 μm. (J) The relative areas of an open wound in (I) were quantified and plotted. (K) The proliferation assay of HTR8/SVneo cells measured by EdU assay. (L) Quantitative analysis of proliferation in (K) (*p < 0.05; **p < 0.01; ***p < 0.001).

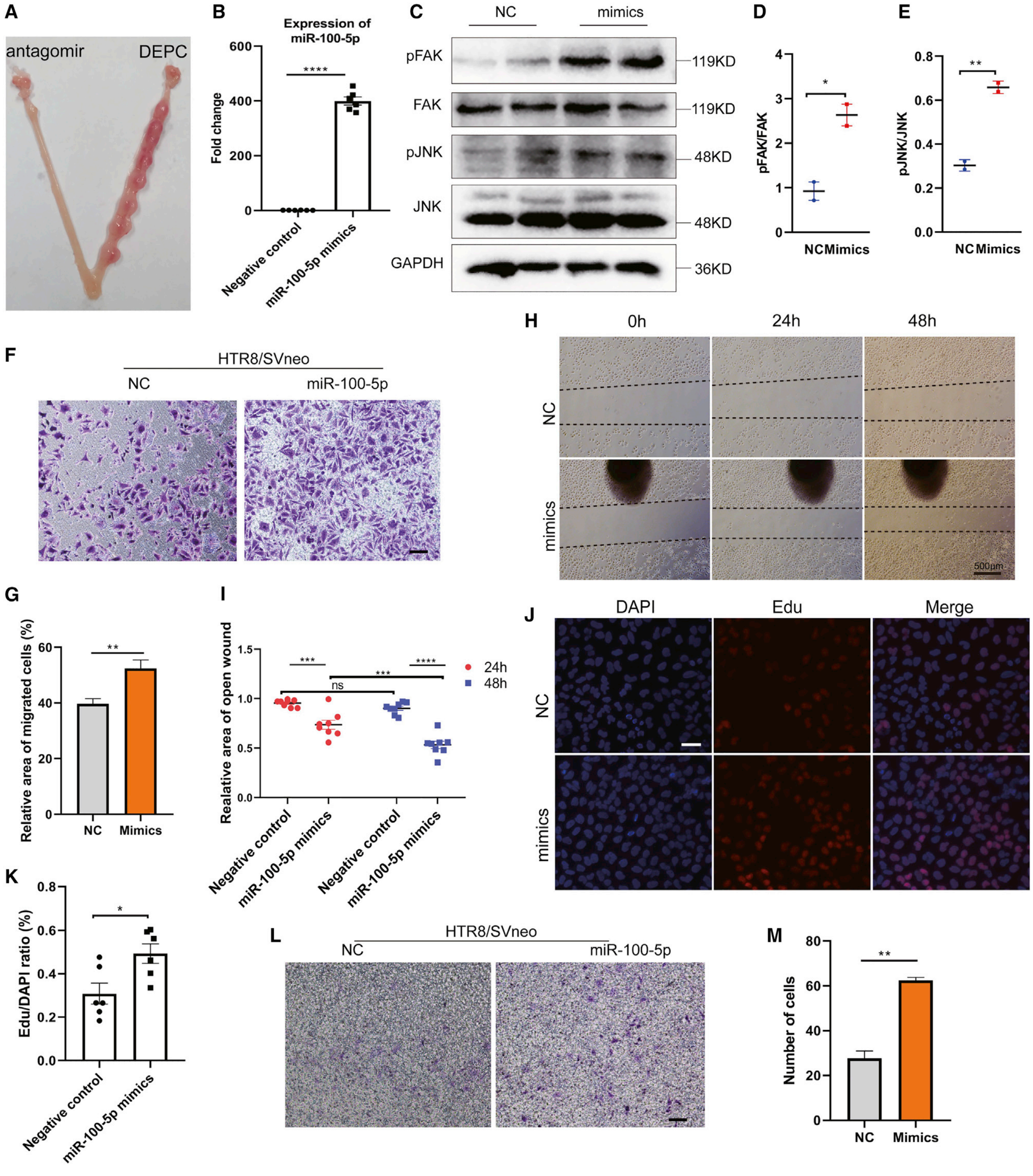

Figure 5. Exosomal miR-100-5p promotes the ability (migration, invasion, and proliferation) of trophoblasts for embryo implantation

(A) Day 7 implantation sites of females after injecting miR-100-5p antagomir or DEPC water in each side of horn and arrows indicate implanted embryos. (B) The expression of miR-100-5p in HTR8/SVneo is detected after transfecting miR-100-5p mimics or negative control for 24 h. (C–E) HTR8/SVneo trophoblasts were transfected with miR-100-5p mimics or negative control. 24 h later, the cells were immunoblotted for P-FAK and P-JNK. The blots were also detected for total FAK, JNK, and GAPDH. The ratio of phospho protein was measured and calculated using phospho protein/total protein. (F and G) Migration assay of HTR8/SVneo cell-transfected miR-100-5p-mimic or negative control. Relative areas of migrated cells were counted, and representative images were shown. Bar, 50 μm. (H) Wound-closure assays were performed on

(legend continued on next page)

in Figure 5A, inhibition of miR-100-5p during WOI drastically impeded the ability of embryo implantation (Figure 5A, left uterine horns) compared to the DEPC-injected group. miRNAs encapsulated in exosomes are abundant, and the exosomes derived from the uterus play an important role in successful embryo implantation.[9] To confirm that this is the case, we transfected miR-100-5p mimics into HTR8/SVneo cells (Figure 5B). After 24 h, we examined the P-FAK and P-JNK levels. Consistent with the HTR8/SVneo trophoblast results after stimulation with exosomes, we found that miR-100-5p dramatically phosphorylated FAK or JNK compared to the control (Figures 5C–5E). Furthermore, we set out to determine whether miR-100-5p promotes the ability of migration of HTR8/SVneo trophoblasts. The results from the Transwell assay confirmed that miR-100-5p enhanced the migration of HTR8/SVneo cells (Figures 5F and 5G). In addition, wound-healing assays also reflected that miR-100-5p mimics promote the edges healing, and the effect increased with prolonged transfection time (Figure 5H). However, there was no significant difference in the wound healing in the control group, 24 h or 48 h after transfection, but the cells significantly migrated after treatment with miR-100-5p mimics (Figure 5G). Collectively, these results indicated that miR-100-5p promotes the migration of HTR8/SVneo trophoblasts. Moreover, we found that miR-100-5p mimics contributed to the proliferation potential of HTR8/SVneo cells (Figure 5J). The miR-100-5p transfection group had high proliferation of HTR8/SVneo trophoblasts compared to the control group (Figure 5K). Additionally, as we expected, the Transwell invasion assay revealed that miR-100-5p mimics promote the invasion of HTR8/SVneo cells (Figures 5L and 5M). Overall, the study highlights the importance of endometrial cell-derived sEV miR-100-5p in activating FAK or JNK. The activation of FAK or JNK signaling mediates the migration and invasion ability of trophoblasts that is required for embryo implantation.

sEV-derived miR-100-5p promotes angiogenesis

Angiogenesis, an essential aspect of interaction in the maternal-embryo interface, is also required for successful embryo implantation. Angiogenesis and increased vascular permeability during implantation could provide a way of communication between the maternal uterus and embryos. Besides, it maintains the maternal-embryo interface that nourishes the environment.[30] Endothelial cells have the ability to divide and rapidly migrate in response to angiogenic signals. Therefore, we hypothesized that sEVs derived from the endometrium during WOI act as angiogenic signals during embryo implantation and development. Initially, the ability of migration of human umbilical vein endothelial cells (HUVECs) was determined using the receptive EEC-derived sEVs. We found that Ishikawa-derived sEVs promoted the migration of HUVECs compared to the control group (Figures 6A, left panel, and 6B). In addition, we questioned whether sEV-derived miR-100-5p (abundant in Ishikawa-sEVs) plays a role in angiogenesis. The wound-healing assay showed that HUVECs transfected with miR-100-5p mimics exhibited increased cell migration (Figures 6A, right panel, and 6C). On the other hand, to examine the effect of sEV-derived miRNA in HUVEC proliferation, HUVECs were incubated with sEVs or transfected with miR-100-5p mimics for 24 h. The same outcomes were observed in HUVEC proliferation after treatment with sEVs or miR-100-5p, respectively. The EdU assay showed that sEV miR-100-5p remarkably promotes the proliferation of HUVECs (Figures 6D–6F). Further, a tube-formation assay was employed to confirm the promotion of angiogenesis by sEV miR-100-5p. After treatment with sEV or miR-100-5p mimics, HUVECs were allowed to grow for 6 h in a 48-well plate, which was precoated with Matrigel. In brightfield microscopy, the results showed that HUVECs incubated with Ishikawa-sEVs or transfected with miR-100-5p mimics had a higher tendency toward ring formation than control groups (Figures 6G and 6H). Overall, these results suggest that sEV miR-100-5p derived from receptive EECs positively impacts the angiogenesis of the HUVECs during implantation.

DISCUSSION

Recent research has focused on EVs as novel mechanisms for cell-cell communications, as well as their extensive use as biomarkers in many diseases.[16,17,31] Exosomes are one of the major types of EVs, and they contain cargo that could be transferred from donor cells to recipient cells, thus triggering the signaling and phenotypic shifts in the recipient cells.[13,16,32] Studies have demonstrated the critical roles of sEVs in numerous physiological processes, such as tumor progression,[33] immune response,[34] and embryo development, in early pregnancy.[35] For example, EVs derived from embryonic stem cells regulate trophoblast behavior during the implantation process.[28] Other studies have demonstrated that uterine cells secrete sEVs that influence the embryonic development during pregnancy.[36] Whereas most of the studies have focused on the secretion of the uterus and the contents of sEVs derived from uterine cells, exosomal mechanisms in receptive phases of embryo implantation are not available.

Embryo implantation is an essential step in the development of a pregnancy, where embryo establishes contact with the receptive uterus, including adhesion, invasion, and angiogenesis.[4,6] In this study, we explored the mechanisms of action of sEVs in embryo implantation during WOI. We have shown that receptive EECs contain more MVBs and ILVs than nonreceptive EECs, and sEVs get secreted from the EECs into the culturing media. Results from NTA and protein concentration showed that unlike the nonreceptive EECs, receptive EECs generate more particles. We further showed that EEC sEVs transfer their contents to trophoblasts, and when injected into the uterus in the early stage of pregnancy, nonreceptive EEC sEVs

HTR8/SVneo cells of transfection with miR-100-5p mimics or negative control. The images were captured in 24 h and 48 h after transfection. Bar, 500 μm. (I) Relative area of open wound in (H) was quantified and plotted. (J and K) HTR8/SVneo cells were transfected directly with miR-100-5p mimics or negative control. Subsequently, after 24 h, the ratio of the cells of proliferation was counted and plotted. Representative images were captured using fluorescence microscopy and shown. (L and M) Invasion analysis of HTR8/SVneo cells transfected with miR-100-5p mimics or negative control. Bar, 50 μm. The invaded cells were counted, and representative images were shown (*$p < 0.05$; **$p < 0.01$; ***$p < 0.001$).

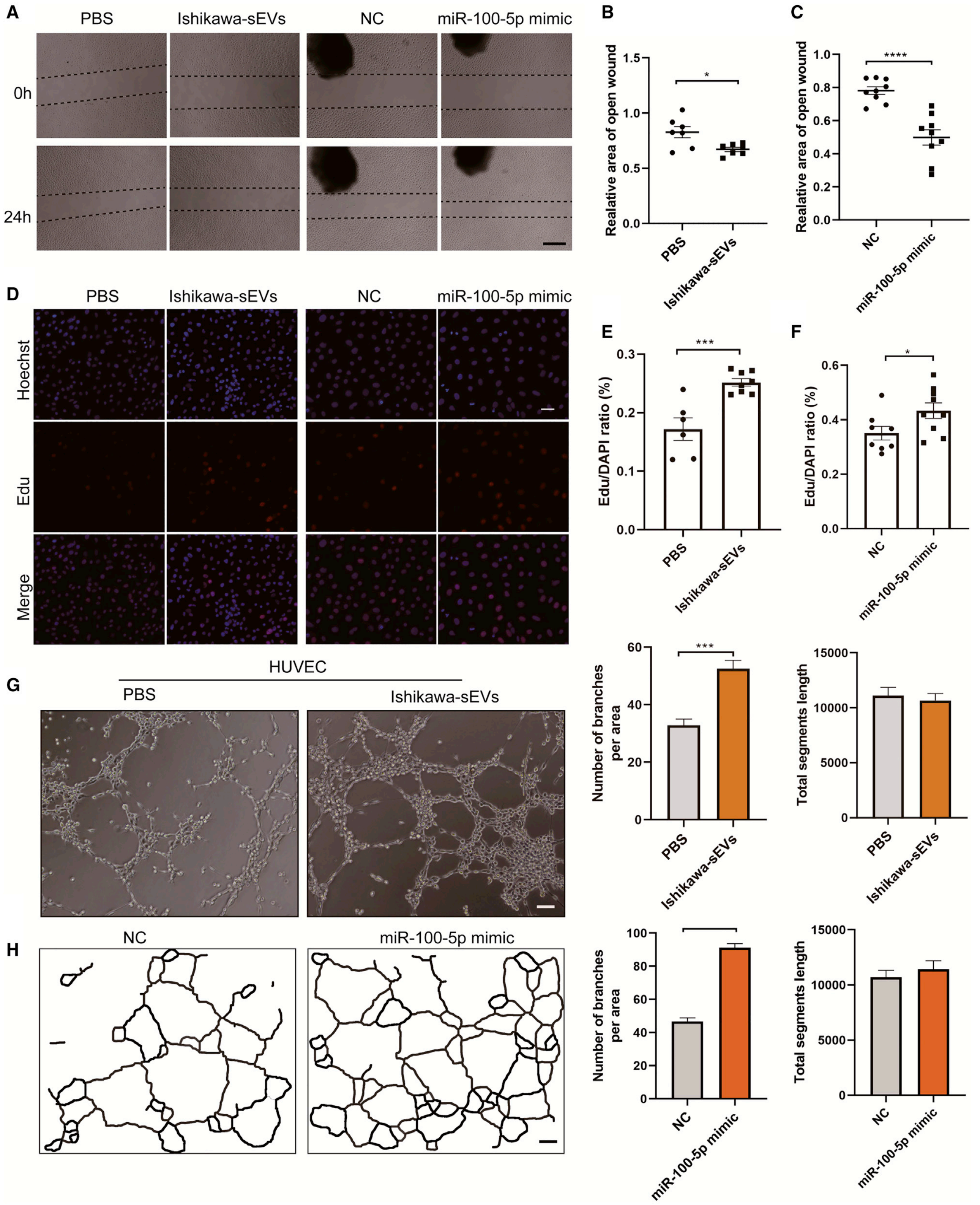

(legend on next page)

markedly decrease the number of implanted embryos. Therefore, the findings suggest that endometrial cells release more sEVs to support embryo implantation during WOI. In addition, EEC-derived sEVs were directly taken up by trophoblasts. It is precisely because sEVs derived from receptive endometrial cells are transported to trophoblast cells that FAK and JNK kinases in the cells are activated, which in turn, affects cell functions. The activation of the kinases enhances trophoblast migration, invasion, and proliferation. Receptive EEC-derived sEVs also contribute to angiogenesis during WOI. However, nonreceptive EEC sEVs do not affect trophoblasts. These findings provide, for the first time, evidence that sEVs affect embryo implantation.

Previous studies have shown that sEVs are the main source of circulating miRNAs and that the exosomal miRNAs contribute to the progression of some cancers. sEVs enrich miRNAs selectively with specific physiological or pathological processes.[37] In mammals, miRNAs play an essential role in the preparation of uterine receptivity and successful pregnancy.[22,38] To further understand which of the sEV miRNAs are responsible for the enhancement of trophoblast potency in implantation, we sequenced miRNAs from Ishikawa-sEVs or HEC-1-A-sEVs. In analyzing the DEMs, miR-100-5p was noticed because it mainly enriches receptive EEC sEVs, and it has been shown that miR-100-5p could promote the migration of cancer cells.[39] We observed that miR-100-5p is highly expressed in endometrium during WOI, and the inhibition of miR-100-5p affects the normal embryo implantation. At present, there are few studies on the role of miR-100-5p in the reproduction process, especially in the stage of embryo implantation. However, some studies have shown that miR-100-5p inhibits the occurrence of preeclampsia in pregnancy, indicating that miR-100-5p plays an important role in the maintenance of normal pregnancy.[40] In different stages of early pregnancy (preimplantation, implantation, and postimplantation), uterine secretions (uterine fluid) have different miRNA expression profiles. Moreover, the transfer of these miRNAs to the embryo affects the embryonic transcriptome and regulates the development and attachment of early embryos.[23] In the present study, the transfection experiments showed that miR-100-5p directly promotes the migration, invasion, and proliferation of trophoblasts, as well as contributes to the angiogenesis. sEV-derived miR-100-5p was shown to stimulate P-FAK and P-JNK in trophoblasts. These findings indicate that during the implantation window, sEV-derived miR-100-5p from endometrial cells regulates the gene expression and signaling pathway, thus affecting the function of trophoblasts cells. Current studies have shown that in endometriosis, miR-100-5p is highly expressed and promotes invasion by inhibiting the expression of SMARCD1,[41] indicating the function of miR-100-5p. In our research, we speculate that the sEV miR-100-5p is internalized by embryonic trophoblast cells and targets the gene expression of trophoblast cells. Based on the previous pathway enrichment analysis of sEV miRNA target genes and the activation of FAK and JNK by miR-100-5p, we considered that miR-100-5p is most likely to participate in focal adhesion and PI3K/AKT signaling pathways, thereby regulating cell migration and invasion. Overall, our study uncovers, for the first time, the role of miR-100-5p in uterine physiology and shows the role of the exosomal miR-100-5p in mediating efficient embryo implantation. However, the specific molecular mechanisms of sEV miR-100-5p downstream need to be further studied.

Besides the infertility cases, millions of women in the world experience early pregnancy losses, and most of the pregnancy failure is associated with lack of proper contact between maternal uterus and embryos during the time of implantation.[8,42] Although assisted reproduction technology (ART) has achieved some progress, lack of understanding of how the interaction between uterus and embryos is established reduces the rates of pregnancy.[43,44] Our study shows that receptive EEC-derived exosomal miR-100-5p enhances the potency of trophoblasts to implant. We are proposing a new way of understanding the embryo implantation mediated by exosomes. Furthermore, these findings also promote the use of infertility and implantation failure therapies so as to support embryo development as well as regulation of the uterine microenvironment to establish a successful pregnancy.

MATERIALS AND METHODS

Antibodies and main reagents

The sources and usages of antibodies were as follows: CD63 (cat. no. A5271) and CANX (cat. no. A0803) were from ABclonal Technology and diluted at 1:1,000. TSG101 (cat. no. 14497-1-AP) and alpha tubulin (cat. no. 11224-1-AP) were from Proteintech and used at 1:1,000. Glyceraldehyde 3-phosphate dehydrogenase (GAPDH; cat. no. db106), MUC1 (cat. no. db545), Alix (cat. no. db3856), HSP70 (cat. no. db2396), and CD9 (cat. no. db919) were from Diagbio and used at 1:1,000. In addition, anti-MUC1 was used at 1:100 for immunofluorescence detection. Antibodies for recognizing total FAK and JNK or P-FAK (cat. nos. db4203 and db2584) and P-JNK (cat. nos. db58 and db2618) were from Diagbio and used at 1:1,000. The secondary goat anti-rabbit antibodies were used for western blot (WB; ABclonal Technology; 1:3,000) and immunofluorescence (Beyotime; 1:1,000). DiI dye and fluorescein isothiocyanate (FITC)-conjugated

Figure 6. sEV-derived miR-100-5p promotes angiogenesis

(A) Wounds created when HUVECs grew to the proper density. Then, HUVECs were incubated with sEVs derived from Ishikawa cells or transfected with miR-100-5p mimics and allowed to migrate for 24 h. Bar, 500 μm. (B and C) The relative area of the open wound was counted and plotted after treatment with sEV or miRNA mimics, respectively. (D) HUVECs were stimulated with Ishikawa-sEVs or miR-100-5p mimics for 24 h, and the proliferation of cells was examined using the EdU assay. Bar, 50 μm. (E and F) The proliferation rate of HUVECs in (D) was counted and plotted. (G) Representative pictures of tube formation were shown (treatment with PBS or Ishikawa-sEVs). Number of branches and total element lengths were quantified. Bar, 50 μm. (H) Tube-formation assay in HUVECs was performed after treatment with miR-100-5p mimics. The schematic diagram of representative tubes was obtained using Angiogenesis Analyzer for ImageJ. The angiogenesis was quantified and counted. Bar, 50 μm (*$p < 0.05$; **$p < 0.01$; ***$p < 0.001$).

phalloidin were from Beyotime (cat. no. C1036) and Solarbio (cat. no. CA1620), respectively. miRNA mimics and agomirs were from GenePharma (PR China). Other reagents for western blot and cell culture were from Beyotime and Gibco, respectively.

Cell culture

Human EEC lines (Ishikawa and HEC-1-A) were purchased from the cell bank of the Chinese Academy of Science (Shanghai, PR China). HTR8/SVneo trophoblast cells were purchased from ATCC. HUVEC was a gift from Dr. Qin Zhiyuan, College of Pharmaceutical Sciences, Zhejiang University. Ishikawa cells and HUVECs were cultured in DMEM medium (Gibco), supplemented with 10% (v/v) FBS (Gibco). HEC-1-A cells were cultured in McCoy's 5A (Sigma), supplemented with 10% (v/v) FBS. HTR8/SVneo cells were cultured in RPMI-1640 medium (Gibco), supplemented with 5% (v/v) FBS.

Isolation and purification of sEVs

For sEV purification from cell culture medium, cells were cultured with 10% exosome-free FBS. Exosome-depleted FBS was prepared by overnight ultracentrifugation at 100,000 × *g*, 4°C. Cells were cultured for 48–36 h, and then supernatants were collected for sEV isolation using a standard centrifugation protocol, which referenced previous research. In brief, cell culture supernatants were centrifuged at 500 × *g* for 10 min and 2,000 × *g* for 20 min to remove dead cells and debris. Following, the supernatants were centrifuged at 10,000 × *g* for 30 min and filtered with 0.22 μm to pellet big microvesicles. Supernatants were then centrifuged at 120,000 × *g* for 75 min twice. The pelleted sEVs were suspended in PBS for further usages.

The size distribution and concentration of sEVs isolated from cell culture supernatants were determined using a ZetaView PMX 100 (Particle Metrix, Meerbusch, Germany). For each group, the analysis was performed independently at least three times.

Transmission electron microscopy

10 μL of freshly isolated sEVs was transferred onto formvar carbon-coated copper grids to dry, rinsed in double-distilled water, and negatively stained by 2% uranyl acetate at room temperature for 1 min. The image was observed with a Tecnai G2 Spirit 120 kV transmission electron microscope operating at 120 kV (Thermo Fisher Scientific, FEI).

For observation of MVBs in cells, Ishikawa and HEC-1-A cells were harvested, centrifuged, and washed with PBS. The cell mass was fixed with 2.5% glutaraldehyde overnight at 4°C and rinsed in water. Following, the cells were fixed with 1% osmic acid, stained using 2% uranyl acetate, and dehydrated in gradient alcohol. Finally, the cell mass was embedded in epoxy resin. Ultrathin sections were prepared, stained with uranyl acetate and lead citrate, and examined by a frozen transmission electron microscope.

Confocal microscopy

For the characterization of Ishikawa and HEC-1-A cell lines, MUC1 in cells was detected using immunofluorescence. In brief, 50% confluent cells were washed with PBS and fixed with 4% paraformaldehyde for 20 min at room temperature. Then, the cells were treated with 0.1% Triton X-100 and blocked with 5% bovine serum albumin (BSA). The cells were incubated with MUC1 antibody, washed, and then incubated with A555-conjugated secondary antibody, FITC-conjugated phalloidin, and 4′,6-diamidino-2-phenylindole (DAPI; Beyotime). Cells were rinsed with PBS before being measured by fluorescence microscopy. To visualize leading edges on HTR8/SVneo, cells, which had been stimulated with exosomes, were fixed with 4% paraformaldehyde, permeabilized with 0.1% Triton X-100, blocked with 5% BSA, and stained with DAPI. Then, cells were visualized by a Zeiss laser-scanning confocal microscope (Germany). Images were captured by ZEN 2010 software.

For uptake tracking analysis, 50 μg sEVs was incubated with DiI dye with 20 μM. sEVs were purified by ultracentrifugation at 10,0000 × g. Purified sEVs were then added to the HTR8/SVneo cell culture and incubated for 2, 4, 6, and 12 h. Cells were washed and fixed. Then, cells were treated with 0.1% Triton X-100 and stained with DAPI. Confocal microscopy was used for visualization.

Western blot

WCLs or sEV proteins were separated using 10%–12% SDS-PAGE gels and transferred to polyvinylidene difluoride membranes (Millipore). The blots were blocked with QuickBlock western buffer (Beyotime) for 20 min at room temperature and incubated with primary antibody at dilutions recommended by the manufacturer at 4°C overnight. Following, the blots were incubated with horseradish peroxidase (HRP)-conjugated secondary antibodies at room temperature for 2 h. Immunodetection was detected using BeyoECL Plus (Beyotime; cat. no. P0018S). CD63, Alix, TSG101, HSP70, and CD9 were used as exosome markers. CANX was used as a negative control. MUC1 was used to distinguish cell lines. GAPDH and alpha tubulin were used as a loading control.

Transwell assay

Ishikawa and HEC-1-A cells were incubated with 10 μM DiI (Beyotime) for 20 min at room temperature. For coculture assay, HTR8/SVneo cells were plated in 12-well Transwell plates with inserts (Corning). DiI-labeled Ishikawa and HEC-1-A cells were seeded into the upper compartment of inserts. Then, HTR8/SVneo cells were cocultured with labeled cells for 12 h. Fluorescence detection was performed using a fluorescence microscope (Nikon).

Cell proliferation assay

Cells were incubated with 10 μM EdU (cat. no. C0075S; Beyotime) in a 12-well plate for 2 h and fixed with 4% paraformaldehyde for 15 min at room temperature. Then, cells were washed with 1 mL PBS, 3 times, and permeabilized using PBS containing 0.3% Triton X-100 for 15 min. Next, a 200-μL click reaction mixture (Beyotime) was added to each well and incubated for 30 min. Hoechst was used to stain the nucleus. Images were captured using a fluorescence microscope (Nikon).

RNA extraction and qRT-PCR assay

The same amount of *Caenorhabditis elegans* cel-39-3p miRNA was spiked into each sEV sample as an external calibration for RNA extraction, RT, and miRNA amplification. Total RNAs were extracted from cells using the Trizol reagent (Tiangen; cat. no. DP421). cDNAs were synthesized using the first-strand cDNA synthesis kit (Tiangen; cat. no. KR118). The relative expression of mRNA was measured using the SuperReal PreMix Color (SYBR Green) qRT-PCR Kit (Tiangen; cat. no. FP215). miRNAs in exosomes, cells, and tissues were extracted using the miRNeasy Mini Kit (QIAGEN; cat. no. 217184), according to the manufacturer's protocols. Then, RNA was reverse transcribed to cDNA following the kit protocol (Tiangen; cat. no. KR211). The miRNA level was detected using the miRcute Plus miRNA qPCR Kit (SYBR Green) (Tiangen; cat. no. FP411). Data were normalized to levels of GAPDH (mRNA), U6 (cellular and tissue miRNA), or cel-39 (exosomal miRNAs) and analyzed by the $2^{-\Delta\Delta Ct}$ method.

Exosomal miRNA sequencing (miRNA-seq) and bioinformatics analysis

miRNA components in Ishikawa-sEVs (n = 3) and HEC-1-A-sEVs (n = 3) were profiled by miRNA sequencing analysis (Illumina HiSeq). miRNA sequencing reads were normalized and quality assessed with fastp; then, the clean reads were mapped to reference genomes using miRdeep2 software. Next, the number of miRNA reads in each sample was counted, and RPMs (reads per million) were used to normalize the expression. The limma package was used to estimate precision weights for all observations and then identify DEMs. Visualizations were generated with the ggplot2 and Heatmap R packages. Target genes of DEMs were predicted by miRTarBase (http://mirtarbase.mbc.nctu.edu.tw/php/search.php). Gene Ontology (GO) and Kyoto Encyclopedia of Genes and Genomes (KEGG) analysis were performed using GO packages in R basic.

miRNA transfection

Mimics and antagomir of miR-100-5p were purchased from GenePharma (Shanghai, PR China) and diluted using DEPC water. Cells were cultured in a six-well plate and transfected with miRNAs using the HiPerFect Transfection Reagent (QIAGEN; cat. no. 301702), according to the manufacturer's procedures. In brief, 5 μL of 20 μM miR-100-5p mimics was mixed with transfection reagents and incubated for 10 min at room temperature to allow the formation of transfection complexes. Then, the complexes were added drop-wise onto the cells and incubated for 24 h until analysis.

Cell migration and invasion assay

HTR8/SVneo cells were pretreated with exosomes or miRNA mimics for 24 h before the cell motility assay. Then, a Transwell with polycarbonate membranes (8 μm pore size) (Corning) was employed. For a migration assay, 1×10^5 HTR8/SVneo cells were resuspended with RPMI-1640 medium without FBS and seeded into the inserts of the Transwell. Meanwhile, 500 μL RPMI 1640 containing 10% exosome-free FBS was added to the lower compartment. For invasion assay, the inserts of Transwell were precoated with Corning Matrigel (cat. no. 356234) and incubated at 37°C for 2 h to allow polymerization. Next, the cells were cultured in the upper compartment of Transwell without FBS and placed into the lower chamber with 500 μL RPMI 1640 containing 10% exosome-free FBS. After incubation for 24 h, the cell inserts were fixed and stained with 0.1% crystal violet solution (Solarbio; cat. no. G1064). Representative fields were photographed, and the number of cells was counted.

Wound-closure assays

Cells were plated into six-well plates until 70%–80% cell confluent monolayers. Then, the cells were serum starved and wounded using a pipet tip. After removing shedding cells, cells were treated with EEC-derived sEV or miRNA mimics and allowed to migrate for 24 h. The extent of wound closure was observed under the microscope and analyzed.

Injection test

For implantation site detection, a surgical operation was performed for the female mouse on D3 of pregnancy. Equal quality miR-100-5p antagomir or sEVs and their control were injected into one side of the uterine horn, respectively. 4 days later, the number of implantation sites was checked.

Tube-formation assay of HUVEC

In vitro angiogenesis experiments were determined by performing a tube-formation assay in Matrigel (Corning). 48-well plates were precoated using 200 μL Matrigel and placed in a cell incubator at 37°C for 2 h. Then, HUVECs with treatment (sEV or miRNA mimics) were resuspended in medium, supplemented with 10% FBS, seeded into 48-well plates, and cultured for 8 h. Tube formation was examined by an optical microscope (Nikon), and the branch density and tube length were quantified and plotted.

Statistics

Values in this study were reported as mean ± SEM. The Student's t test (two-tailed) was used to compare the difference between the two groups. One-way ANOVA was performed when more than two treatments were compared. Plots used GraphPad Prism 8.0 or R basic. The sequences of primers, agomir, and miRNA mimics were shown in Table S1. ***p values $\leq$ 0.001, **p values $\leq$ 0.01, *p values $\leq$ 0.05, and ns p values $\geq$ 0.05.

SUPPLEMENTAL INFORMATION

Supplemental Information can be found online at https://doi.org/10.1016/j.omtn.2020.10.043.

ACKNOWLEDGMENTS

This research was supported by grants from the Department of Science Technology of Huzhou City (2019GZ14), Zhejiang Team Science and Technology Commissioner Project (Tongxiang) and Agricultural Science and Technology Innovation Team of Huzhou City (2019HN01).

AUTHOR CONTRIBUTIONS

Q.T. and Z.W. designed the experiments. Q.T. and S.S. mainly conducted the experiments and analyzed the data. Q.T., S.S., and J.L. wrote the paper. D.C. and S.W. were involved with the experiments. Z.W. organized and supervised the study.

DECLARATION OF INTERESTS

The authors declare no competing interests.

REFERENCES

1. Norwitz, E.R., Schust, D.J., and Fisher, S.J. (2001). Implantation and the survival of early pregnancy. N. Engl. J. Med. *345*, 1400–1408.
2. Red-Horse, K., Zhou, Y., Genbacev, O., Prakobphol, A., Foulk, R., McMaster, M., and Fisher, S.J. (2004). Trophoblast differentiation during embryo implantation and formation of the maternal-fetal interface. J. Clin. Invest. *114*, 744–754.
3. Achache, H., and Revel, A. (2006). Endometrial receptivity markers, the journey to successful embryo implantation. Hum. Reprod. Update *12*, 731–746.
4. Cha, J., Sun, X., and Dey, S.K. (2012). Mechanisms of implantation: strategies for successful pregnancy. Nat. Med. *18*, 1754–1767.
5. Daikoku, T., Cha, J., Sun, X., Tranguch, S., Xie, H., Fujita, T., Hirota, Y., Lydon, J., DeMayo, F., Maxson, R., and Dey, S.K. (2011). Conditional deletion of Msx homeobox genes in the uterus inhibits blastocyst implantation by altering uterine receptivity. Dev. Cell *21*, 1014–1025.
6. Wang, H., and Dey, S.K. (2006). Roadmap to embryo implantation: clues from mouse models. Nat. Rev. Genet. *7*, 185–199.
7. Karizbodagh, M.P., Rashidi, B., Sahebkar, A., Masoudifar, A., and Mirzaei, H. (2017). Implantation Window and Angiogenesis. J. Cell. Biochem. *118*, 4141–4151.
8. Wilcox, A.J., Baird, D.D., and Weinberg, C.R. (1999). Time of implantation of the conceptus and loss of pregnancy. N. Engl. J. Med. *340*, 1796–1799.
9. Machtinger, R., Laurent, L.C., and Baccarelli, A.A. (2016). Extracellular vesicles: roles in gamete maturation, fertilization and embryo implantation. Hum. Reprod. Update *22*, 182–193.
10. Fang, T., Lv, H., Lv, G., Li, T., Wang, C., Han, Q., Yu, L., Su, B., Guo, L., Huang, S., et al. (2018). Tumor-derived exosomal miR-1247-3p induces cancer-associated fibroblast activation to foster lung metastasis of liver cancer. Nat. Commun. *9*, 191.
11. Czystowska-Kuzmicz, M., Sosnowska, A., Nowis, D., Ramji, K., Szajnik, M., Chlebowska-Tuz, J., Wolinska, E., Gaj, P., Grazul, M., Pilch, Z., et al. (2019). Small extracellular vesicles containing arginase-1 suppress T-cell responses and promote tumor growth in ovarian carcinoma. Nat. Commun. *10*, 3000.
12. Luo, A., Zhou, X., Shi, X., Zhao, Y., Men, Y., Chang, X., Chen, H., Ding, F., Li, Y., Su, D., et al. (2019). Exosome-derived miR-339-5p mediates radiosensitivity by targeting Cdc25A in locally advanced esophageal squamous cell carcinoma. Oncogene *38*, 4990–5006.
13. Yáñez-Mó, M., Siljander, P.R.M., Andreu, Z., Zavec, A.B., Borràs, F.E., Buzas, E.I., Buzas, K., Casal, E., Cappello, F., Carvalho, J., et al. (2015). Biological properties of extracellular vesicles and their physiological functions. J. Extracell. Vesicles *4*, 27066.
14. Shao, H., Im, H., Castro, C.M., Breakefield, X., Weissleder, R., and Lee, H. (2018). New Technologies for Analysis of Extracellular Vesicles. Chem. Rev. *118*, 1917–1950.
15. Tkach, M., and Théry, C. (2016). Communication by Extracellular Vesicles: Where We Are and Where We Need to Go. Cell *164*, 1226–1232.
16. Mathieu, M., Martin-Jaular, L., Lavieu, G., and Théry, C. (2019). Specificities of secretion and uptake of exosomes and other extracellular vesicles for cell-to-cell communication. Nat. Cell Biol. *21*, 9–17.
17. Théry, C. (2015). Cancer: Diagnosis by extracellular vesicles. Nature *523*, 161–162.
18. Colombo, M., Raposo, G., and Théry, C. (2014). Biogenesis, secretion, and intercellular interactions of exosomes and other extracellular vesicles. Annu. Rev. Cell Dev. Biol. *30*, 255–289.
19. Zhang, X., Sai, B., Wang, F., Wang, L., Wang, Y., Zheng, L., Li, G., Tang, J., and Xiang, J. (2019). Hypoxic BMSC-derived exosomal miRNAs promote metastasis of lung cancer cells via STAT3-induced EMT. Mol. Cancer *18*, 40.
20. Staun-Ram, E., and Shalev, E. (2005). Human trophoblast function during the implantation process. Reprod. Biol. Endocrinol. *3*, 56.
21. Ying, W., Riopel, M., Bandyopadhyay, G., Dong, Y., Birmingham, A., Seo, J.B., Ofrecio, J.M., Wollam, J., Hernandez-Carretero, A., Fu, W., et al. (2017). Adipose Tissue Macrophage-Derived Exosomal miRNAs Can Modulate In Vivo and In Vitro Insulin Sensitivity. Cell *171*, 372–384.e12.
22. Paul, A.B.M., Sadek, S.T., and Mahesan, A.M. (2019). The role of microRNAs in human embryo implantation: a review. J. Assist. Reprod. Genet. *36*, 179–187.
23. Vilella, F., Moreno-Moya, J.M., Balaguer, N., Grasso, A., Herrero, M., Martínez, S., Marcilla, A., and Simón, C. (2015). Hsa-miR-30d, secreted by the human endometrium, is taken up by the pre-implantation embryo and might modify its transcriptome. Development *142*, 3210–3221.
24. Wang, X., Tian, F., Chen, C., Feng, Y., Sheng, X., Guo, Y., and Ni, H. (2019). Exosome-derived uterine microRNAs isolated from cows with endometritis impede blastocyst development. Reprod. Biol. *19*, 204–209.
25. Brayman, M., Thathiah, A., and Carson, D.D. (2004). MUC1: a multifunctional cell surface component of reproductive tissue epithelia. Reprod. Biol. Endocrinol. *2*, 4.
26. Hannan, N.J., Paiva, P., Dimitriadis, E., and Salamonsen, L.A. (2010). Models for study of human embryo implantation: choice of cell lines? Biol. Reprod. *82*, 235–245.
27. Homer, H., Rice, G.E., and Salomon, C. (2017). Review: Embryo- and endometrium-derived exosomes and their potential role in assisted reproductive treatments–liquid biopsies for endometrial receptivity. Placenta *54*, 89–94.
28. Desrochers, L.M., Bordeleau, F., Reinhart-King, C.A., Cerione, R.A., and Antonyak, M.A. (2016). Microvesicles provide a mechanism for intercellular communication by embryonic stem cells during embryo implantation. Nat. Commun. *7*, 11958.
29. Yang, Q., Gu, W.W., Gu, Y., Yan, N.N., Mao, Y.Y., Zhen, X.X., Wang, J.M., Yang, J., Shi, H.J., Zhang, X., and Wang, J. (2018). Association of the peripheral blood levels of circulating microRNAs with both recurrent miscarriage and the outcomes of embryo transfer in an in vitro fertilization process. J. Transl. Med. *16*, 186.
30. Reynolds, L.P., Killilea, S.D., and Redmer, D.A. (1992). Angiogenesis in the female reproductive system. FASEB J. *6*, 886–892.
31. Min, L., Zhu, S., Chen, L., Liu, X., Wei, R., Zhao, L., Yang, Y., Zhang, Z., Kong, G., Li, P., and Zhang, S. (2019). Evaluation of circulating small extracellular vesicles derived miRNAs as biomarkers of early colon cancer: a comparison with plasma total miRNAs. J. Extracell. Vesicles *8*, 1643670.
32. Théry, C., Zitvogel, L., and Amigorena, S. (2002). Exosomes: composition, biogenesis and function. Nat. Rev. Immunol. *2*, 569–579.
33. Peinado, H., Alečković, M., Lavotshkin, S., Matei, I., Costa-Silva, B., Moreno-Bueno, G., Hergueta-Redondo, M., Williams, C., García-Santos, G., Ghajar, C.M., et al. (2016). Corrigendum: Melanoma exosomes educate bone marrow progenitor cells toward a pro-metastatic phenotype through MET. Nat. Med. *22*, 1502.
34. Poggio, M., Hu, T., Pai, C.C., Chu, B., Belair, C.D., Chang, A., Montabana, E., Lang, U.E., Fu, Q., Fong, L., and Blelloch, R. (2019). Suppression of Exosomal PD-L1 Induces Systemic Anti-tumor Immunity and Memory. Cell *177*, 414–427.e13.
35. Sheller-Miller, S., Trivedi, J., Yellon, S.M., and Menon, R. (2019). Exosomes Cause Preterm Birth in Mice: Evidence for Paracrine Signaling in Pregnancy. Sci. Rep. *9*, 608.
36. Tannetta, D., Dragovic, R., Alyahyaei, Z., and Southcombe, J. (2014). Extracellular vesicles and reproduction-promotion of successful pregnancy. Cell. Mol. Immunol. *11*, 548–563.
37. Ramanathan, S., Shenoda, B.B., Lin, Z., Alexander, G.M., Huppert, A., Sacan, A., and Ajit, S.K. (2019). Inflammation potentiates miR-939 expression and packaging into small extracellular vesicles. J. Extracell. Vesicles *8*, 1650595.
38. Yang, Y., Bai, W., Zhang, L., Yin, G., Wang, X., Wang, J., Zhao, H., Han, Y., and Yao, Y.Q. (2008). Determination of microRNAs in mouse preimplantation embryos by microarray. Dev. Dyn. *237*, 2315–2327.
39. Chen, P., Lin, C., Quan, J., Lai, Y., He, T., Zhou, L., Pan, X., Wu, X., Wang, Y., Ni, L., et al. (2017). Oncogenic miR-100-5p is associated with cellular viability, migration and apoptosis in renal cell carcinoma. Mol. Med. Rep. *16*, 5023–5030.
40. Liu, S., Xie, X., Lei, H., Zou, B., and Xie, L. (2019). Identification of Key circRNAs/lncRNAs/miRNAs/mRNAs and Pathways in Preeclampsia Using Bioinformatics Analysis. Med. Sci. Monit. *25*, 1679–1693.

41. Takebayashi, K., Nasu, K., Okamoto, M., Aoyagi, Y., Hirakawa, T., and Narahara, H. (2020). hsa-miR-100-5p, an overexpressed miRNA in human ovarian endometriotic stromal cells, promotes invasion through attenuation of SMARCD1 expression. Reprod. Biol. Endocrinol. *18*, 31.

42. Clark, A.T. (2019). Human embryo implantation modelled in microfluidic channels. Nature *573*, 350–351.

43. Bashiri, A., Halper, K.I., and Orvieto, R. (2018). Recurrent Implantation Failure-update overview on etiology, diagnosis, treatment and future directions. Reprod. Biol. Endocrinol. *16*, 121.

44. Coughlan, C., Ledger, W., Wang, Q., Liu, F., Demirol, A., Gurgan, T., Cutting, R., Ong, K., Sallam, H., and Li, T.C. (2014). Recurrent implantation failure: definition and management. Reprod. Biomed. Online *28*, 14–38.

OMTN, Volume *23*

Supplemental Information

Endometrial cell-derived small extracellular vesicle miR-100-5p promotes functions of trophoblast during embryo implantation

Qiang Tan, Shuang Shi, Jingjie Liang, Dingren Cao, Shaoyu Wang, and Zhengguang Wang

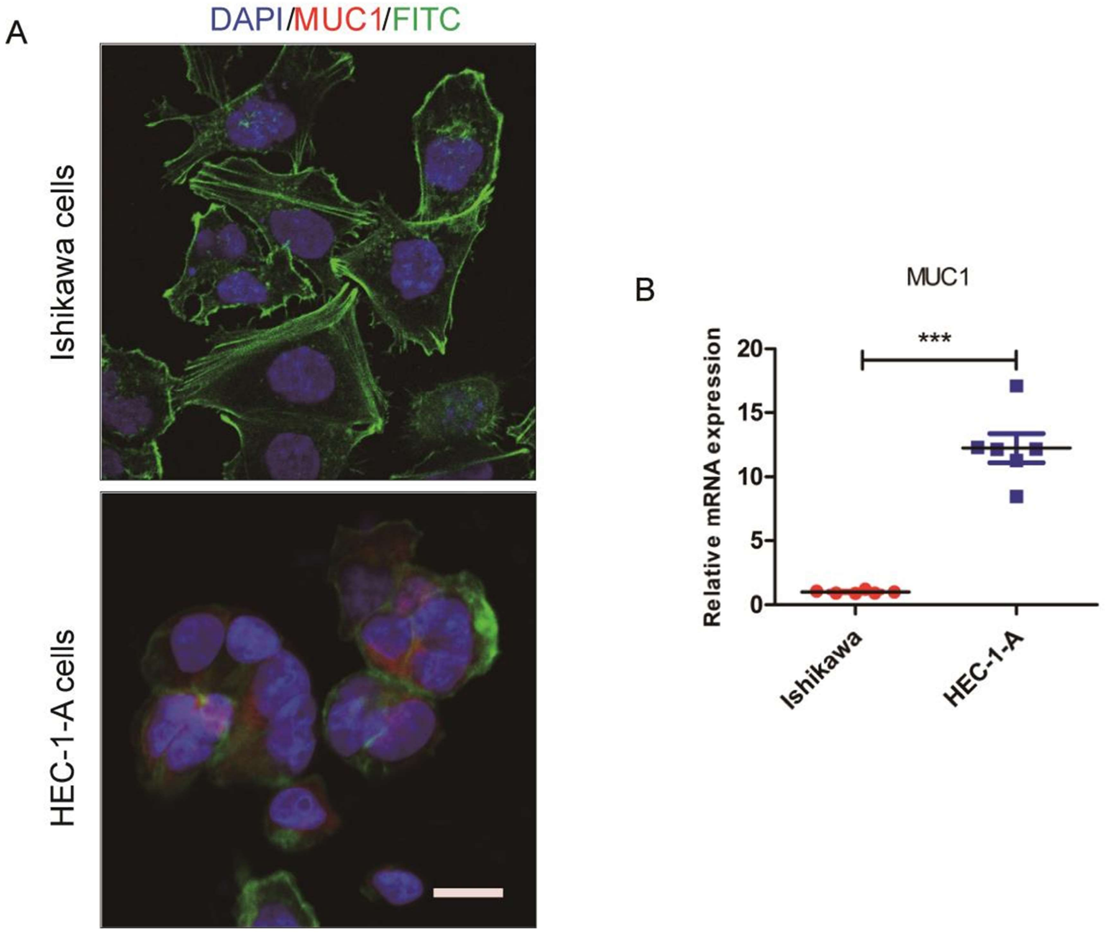

Figure S1. Ishikawa cells and HEC-1-A cells represent receptive and non-receptive endometrium epithelial cells, respectively. (A) Immunofluorescence image of Ishikawa and HEC-1-A cells stained with an MUC1 antibody, a molecular marker of uterine receptivity. Nuclei stained using DAPI dye. FITC-conjugated phalloidin was used to label the cytoskeleton. The scale bar = 20μm. (**B**) The mRNA expression level of MUC1 in Ishikawa and HEC-1-A cells was detected by qRT-PCR. (*P<0.05; **P<0.01; ***P<0.001)

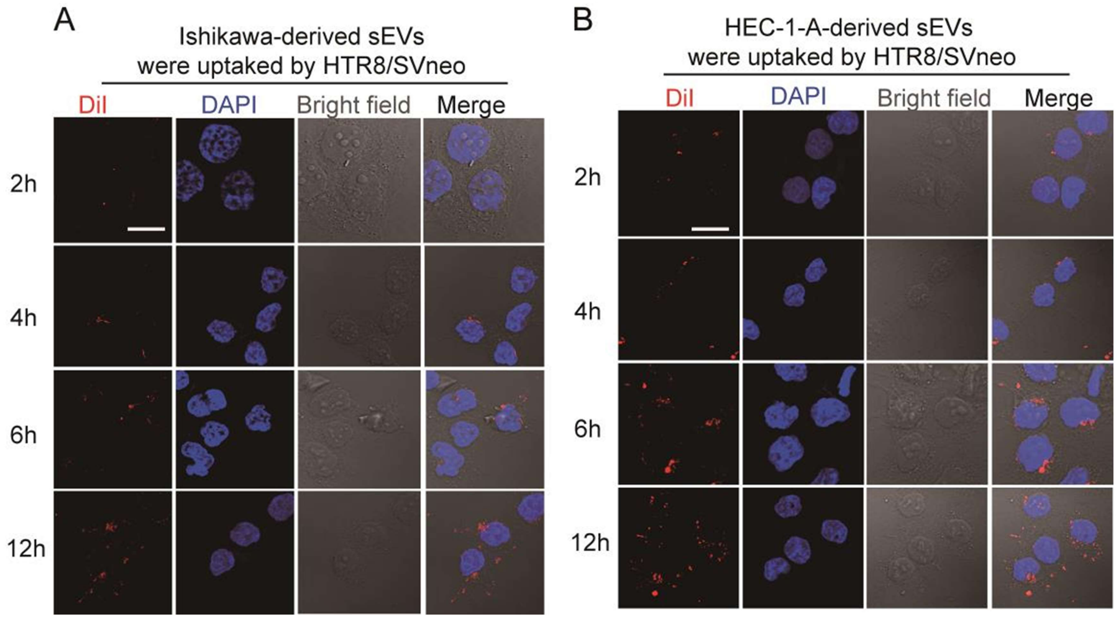

Figure S2. sEVs derived from endometrium epithelial cells could be uptake by HTR8/SVneo trophoblasts. sEVs were labeled using DiI dye and incubated with serum-starved trophoblasts for 2, 4, 6 and 12h, respectively. Confocal microscopic images showed uptake of sEVs derived from Ishikawa cells (**A**) and HEC-1-A cells (**B**) by HTR8/SVneo cells in a time-dependent manner. Nuclei stained by DAPI in blue. The scale bar = 20μm.

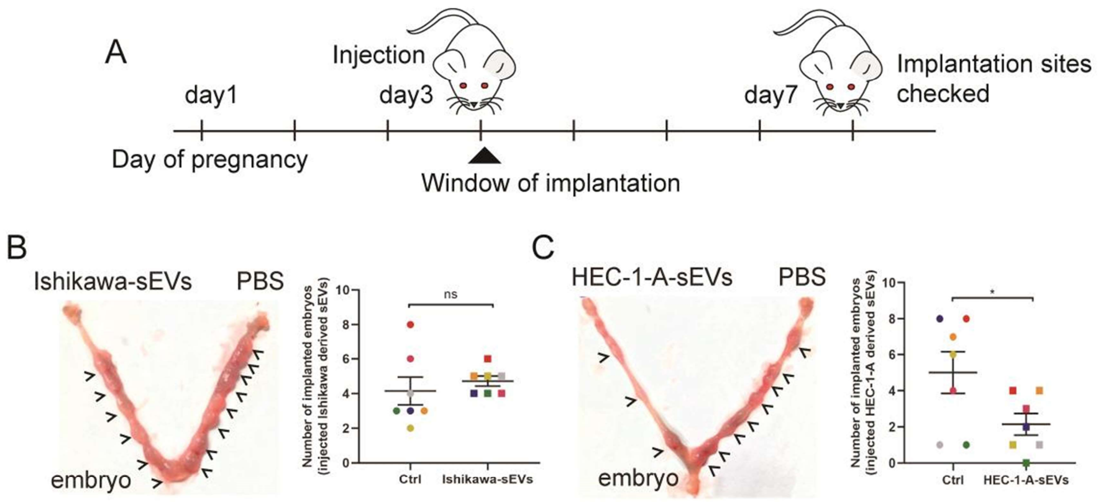

Figure S3. Endometrial epithelial cells derived sEVs impair embryo implantation. (A) Schematic diagram showed the female mouse after mating were injected on day3 of pregnancy, and the number of implanted embryos were checked on day7 of pregnancy. (**B-C)** The number of implanted embryos after injecting Ishikawa or HEC-1-A derived sEVs. PBS was used as a control. (ns: no significant. *P<0.05; **P<0.01; ***P<0.001)

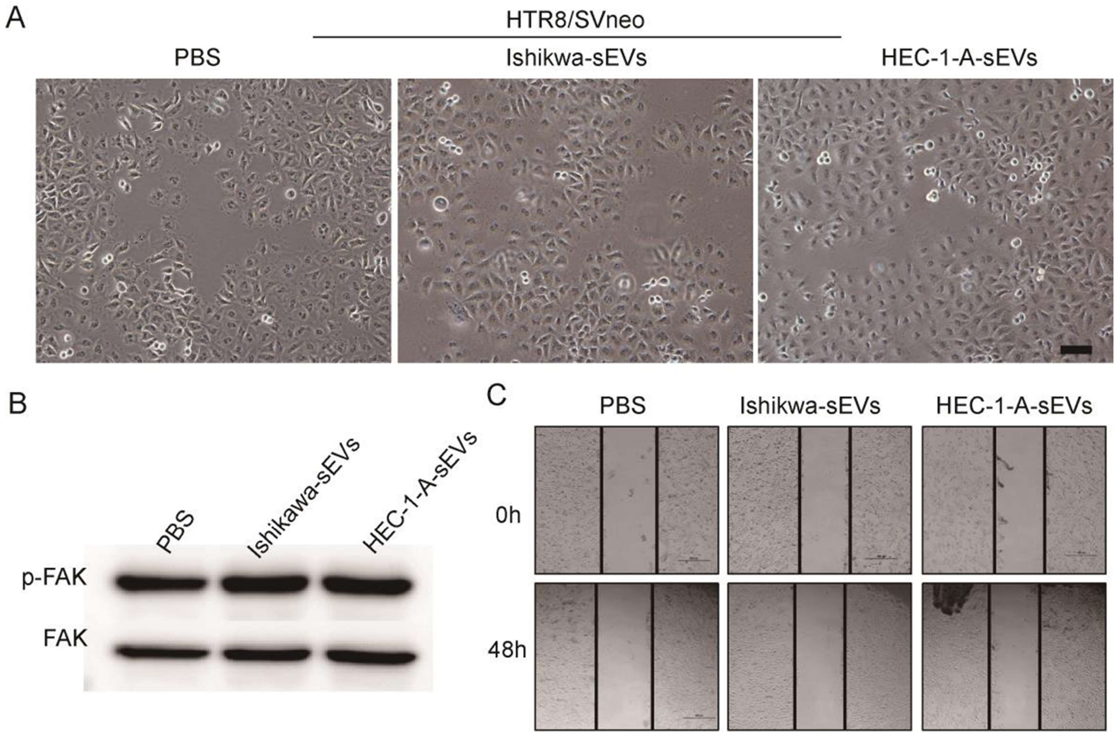

Figure S4. Growing state of HTR8/SVneo cells after stimulation with PBS, Ishikawa- sEVs, or HEC-1-A- sEVs for 24h. (A) The images visualized using the optical microscope. The scale bar = 50μm. (**B**) HTR8/SVneo trophoblasts were serum-starved and treated with PBS, 50μg Ishikawa-sEVs or 50μg HEC-1-A-sEVs. After 48h, the cells were immunoblotted for phosphorylated FAK (P-FAK). (**C)** Images of wound closure assays performed on HTR8/SVneo cells cultured in serum-free medium supplemented PBS, 50μg Ishikawa-sEVs or 50μg HEC-1-A-sEVs for 48h.

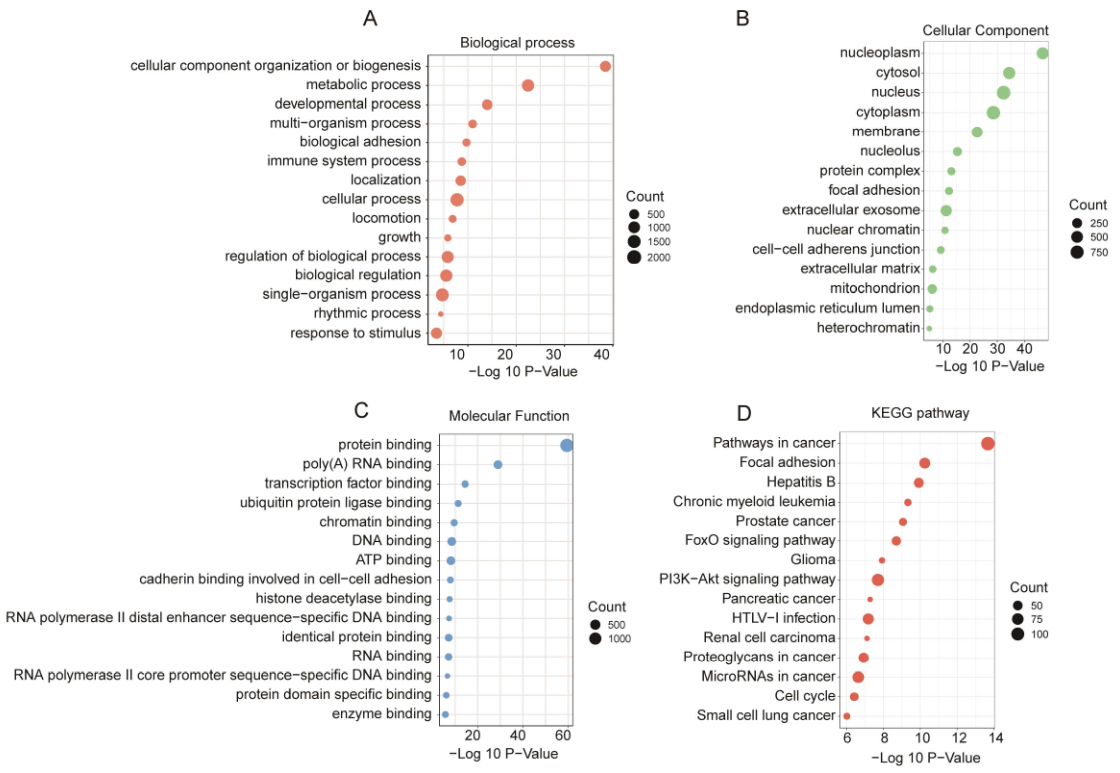

Figure S5. GO and KEGG analysis of genes, which regulated by the differentially expressed miRNAs between Ishikawa-sEVs and HEC-1-A- sEVs. (A-C) A bubble plot of GOs (Biological Process, Molecular function and Cellular components) enriched. (**B**) A bubble plot of KEGG pathway enriched.

Table S1 MiRNA expression profile in endometrial cells derived exosomes (Ishikawa-sEVs vs. HEC-1-A-sEVs)

Table S2 Sequences of primers, agomir, and miRNA mimics.

MUC1	F: 5'TGCCGCCGAAAGAACTACG3'
	R: 5'TGGGGTACTCGCTCATAGGAT3'
GAPDH	F: 5'GGAGCGAGATCCCTCCAAAAT3'
	R: 5'GGCTGTTGTCATACTTCTCATGG3'
miR-100-5p	5'AACCCGTAGATCCGAACTTGTG3'
cel-miR-39	5'TCACCGGGTGTAAATCAGCTTG3'
U6	5'AACGAGAAGCGAACCAAAAAAA3'
miR-100-5p(antagomir)	5'CACAAGUUCGGAUCUACGGGUU3'
miR-100-5p(mimics)	5'AACCCGUAGAUCCGAACUUGUG3'
	5'CAAGUUCGGAUCUACGGGUUUU3'
Negative control (mimics)	5'UUCUCCGAACGUGUCACGUTT3'
	5'ACGUGACACGUUCGGAGAATT3'
